# Nucleic Acid-Based Diagnostic Tests for the Detection SARS-CoV-2: An Update

**DOI:** 10.3390/diagnostics11010053

**Published:** 2021-01-01

**Authors:** Choo Yee Yu, Kok Gan Chan, Chan Yean Yean, Geik Yong Ang

**Affiliations:** 1 Independent Researcher, Kuala Lumpur 51200, Malaysia; chooyee85@gmail.com; 2 Division of Genetics and Molecular Biology, Institute of Biological Sciences, Faculty of Science, University of Malaya, Kuala Lumpur 50603, Malaysia; kokgan@um.edu.my; 3 Department of Medical Microbiology and Parasitology, School of Medical Sciences, Universiti Sains Malaysia, Kota Bharu 16150, Malaysia; 4 Faculty of Sports Science and Recreation, Universiti Teknologi MARA, Shah Alam 40450, Malaysia

**Keywords:** COVID-19, rapid test, RT-PCR, isothermal, lateral flow, LAMP, CRISPR, sequencing, NGS, POC

## Abstract

The coronavirus disease 2019 (COVID-19) caused by severe acute respiratory syndrome coronavirus 2 (SARS-CoV-2) began as a cluster of pneumonia cases in Wuhan, China before spreading to over 200 countries and territories on six continents in less than six months. Despite rigorous global containment and quarantine efforts to limit the transmission of the virus, COVID-19 cases and deaths have continued to increase, leaving devastating impacts on the lives of many with far-reaching effects on the global society, economy and healthcare system. With over 43 million cases and 1.1 million deaths recorded worldwide, accurate and rapid diagnosis continues to be a cornerstone of pandemic control. In this review, we aim to present an objective overview of the latest nucleic acid-based diagnostic tests for the detection of SARS-CoV-2 that have been authorized by the Food and Drug Administration (FDA) under emergency use authorization (EUA) as of 31 October 2020. We systematically summarize and compare the principles, technologies, protocols and performance characteristics of amplification- and sequencing-based tests that have become alternatives to the CDC 2019-nCoV Real-Time RT-PCR Diagnostic Panel. We highlight the notable features of the tests including authorized settings, along with the advantages and disadvantages of the tests. We conclude with a brief discussion on the current challenges and future perspectives of COVID-19 diagnostics.

## 1. Introduction

The World Health Organization (WHO) China Country Office was first alerted to a cluster of pneumonia cases of unknown etiology in late December 2019, marking the beginning of what has come to be known as the coronavirus disease 2019 (COVID-19) [1]. Within a month’s time, a novel betacoronavirus named severe acute respiratory syndrome coronavirus 2 (SARS-CoV-2) was identified as the causative agent, its complete genome sequence was released [2] and standardized laboratory protocols for COVID-19 were developed [3,4,5,6]. Whereas the SARS epidemic in 2003 was effectively brought under control in eight months, the number of new cases and new deaths caused by COVID-19 have continued to soar with over 2.8 million new cases and 39,712 new deaths reported in the week ending on 25 October 2020 [7]. The health care system of a nation can be stretched to capacity and even overwhelmed when there is a rapid rise in COVID-19 cases due to the need for dedicated wards, medical personnel, and substantial use of limited ICU resources [8]. This makes the availability of accurate diagnostic tools for the timely detection of SARS-CoV-2 extremely important so that the isolation of cases, delivery of appropriate patient care and tracing of close contacts can be executed in parallel with the implementation of other non-pharmacological preventive measures to suppress and mitigate the spread of this disease [9,10]. With the complete SARS-CoV-2 genomes released in public databases earlier during the epidemic [11,12], laboratories and commercial in vitro diagnostic (IVD) manufacturers were able to develop their own molecular tests in record time, as by 9 March 2020 more than 200 applications for test performance evaluation were received by the Foundation for Innovative New Diagnostics [13].

This large influx of novel IVDs in the market poses a challenge to the national regulatory agencies (NRAs), particularly in the low- and middle-income countries as they may not have the resources to fulfil all of their core functions at a speed that is required to support the COVID-19 pandemic response [14]. Given that the use of unreliable and unvalidated diagnostics can severely compromise the effectiveness of disease control programs, reliance on the emergency use authorization (EUA) issued by the Food and Drug Administration (FDA) represents an avenue to accelerate the regulatory processes that are needed to make new or unlicensed IVDs available during public health emergencies. As a stringent regulatory authority (SRA) that is widely acknowledged by the international regulatory and procurement community [15], the FDA also works closely with the Centers for Disease Control and Prevention (CDC) to ensure COVID-19 response resources and requirements are addressed. Given that the pandemic has shown no signs of abating, an updated review of the FDA-EUA nucleic acid tests (NATs) is necessary to capture the large outgrowth of technology platforms that have been used to power these tests, particularly as the previous review on this topic only covered up to April 2020 [16].

In nearly a year since the discovery of SARS-CoV-2, tremendous advancement has been seen in the development and commercialization of nucleic acid-based COVID-19 diagnostics. Other than real-time reverse transcription polymerase chain reaction (RT-PCR) tests, sequencing-based diagnostic tests have emerged along with an increasing variation of non-isothermal and isothermal amplification-based tests developed for SARS-CoV-2 testing. In this review, we start with the genomic architecture of SARS-CoV-2 genome which forms the basis of nucleic acid-based diagnostic tests followed by an overview of FDA-EUA NATs. Then we highlight the specimen collection, specimen processing methods and controls to be used in NATs before comprehensive details of each NAT are discussed and summarized. The challenges and future perspective of NAT development including emerging point-of-care (POC) tests are discussed at the end of the review.

## 2. Genomic Architecture and Key Virulence Factors of SARS-CoV-2

In general, coronaviruses (CoVs) are large spherical or pleomorphic, enveloped viruses with distinctive club-shaped projections and harbor unusually large single-stranded, positive-sense, RNA genomes ranging from 26 to 32 kilobases (kb) in length [17,18]. Since the establishment of the *Coronaviridae* family by the International Committee on Taxonomy of Viruses in 1975, the present classification of CoVs recognizes 46 species in 26 subgenera, five genera and two subfamilies that belong to the family *Coronaviridae*, suborder *Cornidovirineae*, order *Nidovirales* and realm *Riboviria* [19]. Among the four genera in the subfamily of *Orthocoronavirinae*, bats are recognized as the major hosts and gene source of alphacoronaviruses and betacoronaviruses, while the gene sources of deltacoronaviruses and gammacoronaviruses are from avian species [20]. Unlike alphacoronaviruses (HCoV-229E and HCoV-NL63) and betacoronaviruses of the A lineage (HCoV-OC43 and HCoV-HKU1) that are associated with common colds and self-limiting upper respiratory tract infections among immunocompetent humans, betacoronaviruses of the B and C lineage (SARS-CoV, SARS-CoV-2 and MERS-CoV) have caused epidemics with a wide spectrum of disease severity [21].

As with other CoVs, the non-segmented genome of SARS-CoV-2 can be readily translated by replicase polyproteins given that the structure resembles that of a typical cellular mRNA with a 5′ cap structure and a 3′ poly(A) tail [17]. The majority of the ~29.9 kb-genome encodes for non-structural proteins (nsps) including the RNA-dependent RNA polymerase (RdRp) that is responsible for viral RNA replication and transcription [12]. The nsp-coding region is more conserved (58% identity) than the structural protein-coding region (43% identity) among different CoV species, suggesting that genetic diversity in the structural proteins is required for adaptation to new hosts [22]. The *Orf1ab*, which is located at the 5′-terminus of the genome, forms the largest open reading frame (ORF) that spanned two-thirds of the whole genome length and gives rise to the production of two large replicase polyproteins (pp1a and pp1ab). A programmed −1 ribosomal frameshifting is responsible for the production of pp1ab as the ribosome will be directed to shift the reading frame by 1 base just upstream of the *Orf1a* termination codon in order to continue the translation of *Orf1ab* [23]. The pp1ab and pp1a are then cleaved by virally encoded proteases into 15 nsps, wherein most of the nsps will become functional components of the replication-transcription complex (RTC) [24].

The remaining one third of the genome at the 3′-terminus encodes for four main structural proteins that are essential for virion assembly and infectivity, namely spike (S), envelope (E), membrane (M) and nucleocapsid (N) proteins. Interspersed between these structural genes are ORFs encoding for eight group-specific accessory proteins. Although accessory proteins are not essential for viral replication, some of these proteins have been shown to be involved in virus-host interactions during CoV infection in vivo and hence contribute to the pathogenicity of the virus [25]. The S, E and M proteins are anchored to the lipid bilayer of the viral envelope and constitute the virus surface proteins. The M protein is the most abundant glycoprotein in the viral envelope and acts as a primary determinant of particle morphology [26]. The E protein only represents a minor component of the viral envelope due to its low copy number but is likely to play a pivotal role, along with the M protein, in virus assembly and budding [27,28]. Although the E and M proteins were shown to be essential for the formation and release of CoV virus-like particles (VLPs) [28], the conflicting results on whether the E protein is required for SARS-CoV pseudoparticle assembly may be attributed to the different cell lines that were used in the studies [29,30,31,32].

In the assembly and secretion of VLPs, the S protein is dispensable but the spikeless virions would be non-infectious [28]. The S protein of SARS-CoV-2 is a trimeric class 1 fusion protein that will be cleaved into S1 and S2 subunits by host proteases [33]. The S1 subunit determines host tropism as it specializes in recognizing and binding to the host cell receptor whereas the S2 subunit mediates the fusion between the viral and cell membranes, leading to the release of the nucleocapsid into the host cell [34]. Similar to SARS-CoV, SARS-CoV-2 utilizes its receptor-binding domain (RBD) in the S1 subunit to interact with the human angiotensin-converting enzyme 2 (ACE2) receptor that is expressed on alveolar epithelial cells and capillary endothelial cells for virus entry [12]. Despite the structural homology in the RBD between SARS-CoV-2 and SARS-CoV (73.9%), the RBD of SARS-CoV-2 exhibits a higher binding affinity for ACE2 due to the greater atomic interactions in SARS-CoV-2-RBD/ACE2 as compared to that of SARS-CoV-RBD/ACE2 [35,36]. Notably absent in SARS-CoV’s S protein is the insertion of four amino acids (PRRA) at the S1/S2 protease cleavage site that results in a furin recognition site: an acquisition that is often found in highly virulent avian and human influenza viruses [37,38]. The presence of a furin recognition site that can be efficiently cleaved was postulated to be advantageous for SARS-CoV-2 by facilitating the conformational change required for RBD exposure that is required to initiate interaction with ACE2 [39]. Consequently, organs with high expression of ACE2 such as the lungs, heart, kidney, bladder and the gastrointestinal tract are highly vulnerable to SARS-CoV-2 infection [40,41].

The core structure inside the envelope is the viral nucleocapsid consisting of genomic RNA and N protein. The N protein plays multiple roles but its primary responsibility is to pack the viral RNA genome into a long helical ribonucleoprotein (RNP) complex called the capsid [42,43]. Besides protecting the genome, the N protein also has regulatory functions in the coronaviral life cycle as in vitro studies have shown that the N protein of SARS-CoV has the ability to interfere with the host cell-cycle cellular machinery [44,45]. Several studies have also demonstrated that the N protein is critical for optimal CoV genomic replication [46,47,48]. During viral assembly and budding, the N protein is vital for incorporating the genomic RNA into progeny viral particles and promotes the formation of complete mature virion [49]. A greater amino acid sequence identity is also shared between the N proteins (90.5%) [42] as compared to the S proteins (~75%) [12,50] of SARS-CoV-2 and SARS-CoV. By virtue of its role in encapsidating the genome, the N protein is one of the predominantly expressed proteins in infected cells. The N and S proteins are highly immunogenic structural peptides of the virus and act as targets for development of COVID-19 diagnostics, therapeutics and vaccines [43].

## 3. FDA-EUA NATs for the Detection of SARS-CoV-2

An accurate diagnosis of COVID-19 cannot be achieved through clinical presentation alone because the clinical signs and symptoms of SARS-CoV-2 infection are not distinctive enough from infections caused by other respiratory viruses and bacteria such as adenovirus, influenza viruses, parainfluenza viruses, respiratory syncytial virus (RSV), rhinovirus, other CoVs, Chlamydia, Legionella, and Mycoplasma [51,52,53,54]. Although virus culture method is generally considered the “gold-standard” for laboratory diagnosis of viral infection, the isolation of SARS-CoV-2 is highly restricted to laboratories with biosafety level 3 facilities [55] and the labor-intensive procedure rarely provides results in a timeframe that is quick enough to influence or impact treatment [56]. SARS-CoV-2 isolation is also not recommended by WHO as a routine COVID-19 diagnostic procedure [57]. Instead, nucleic acid amplification tests (NAATs), such as RT-PCR, are recognized as the standard diagnostic test for the confirmation of COVID-19 by the WHO [57] and CDC [58]. NAAT has become the norm in laboratory diagnosis of viral respiratory tract infection as it circumvents the longer turnaround time of the virus culture method and allows the identification of patients in the early stages of infection through direct detection of the viral genetic material [59].

**Figure 1:**
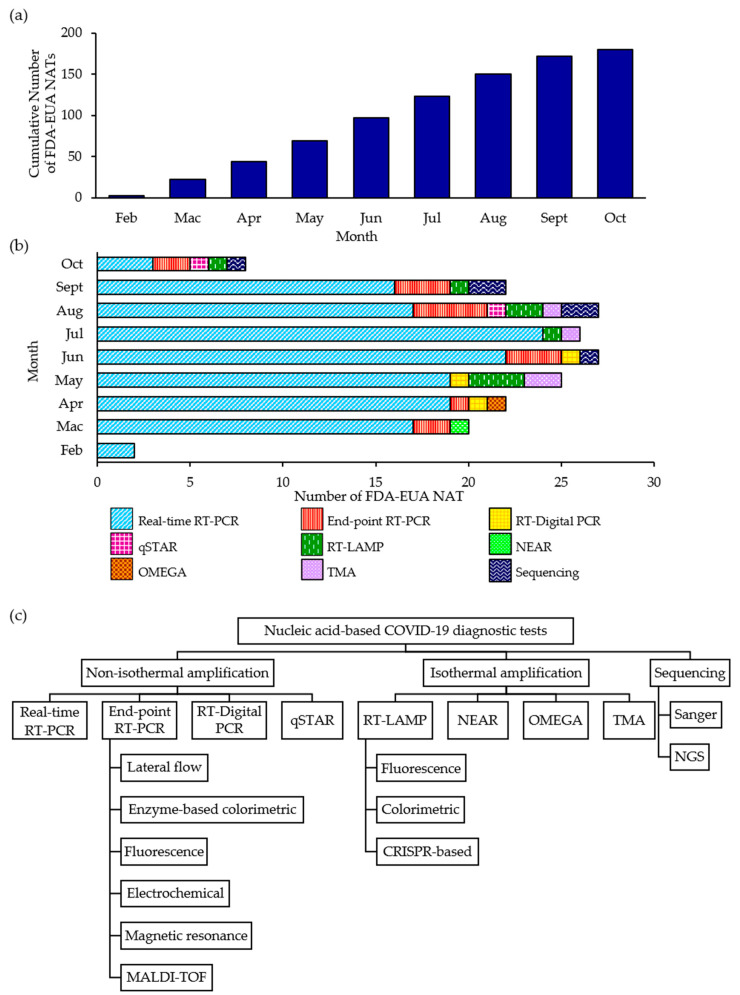
Cumulative number of Food and Drug Administration-emergency use authorization nucleic acid tests (FDA-EUA NATs) (**a**) and the distribution of NATs according to type and month (**b**). An overview of the classification of FDA-EUA NATs in this review (**c**). RT-PCR, reverse transcriptase PCR; MALDI-TOF; matrix-assisted laser desorption ionization-time of flight; qSTAR, Selective Temperature Amplification Reaction; LAMP; loop-mediated isothermal amplification; CRISPR, clustered regularly interspaced short palindromic repeats; NEAR, nicking enzyme amplification reaction; TMA, transcription-mediated amplification; NGS, next generation sequencing.

The discovery of a novel CoV as being responsible for the current pandemic necessitate the development of entirely new IVDs. Through the EUA procedure, a novel or unlicensed diagnostic tool is assessed on whether it can be authorized for use on a time-limited basis after a review is conducted on the documentary evidence submitted by the developer/manufacturer in support of the product’s safety, quality and performance. At the time of writing, a total of 180 NATs has been granted FDA-EUA status (Figure 1a,b) but an EUA may be revised or revoked since authorized tests are still monitored and subjected to the FDA’s continued review of emerging scientific evidence [60]. The FDA-EUA NATs can be broadly divided into three main categories: non-isothermal amplification-based (88.3%), isothermal amplification-based (8.3%) and sequencing-based (3.3%) NATs (Figure 1c). Real-time RT-PCR accounted for 77.2% of the authorized NATs and a large majority of the NATs are limited to Clinical Laboratory Improvement Amendments (CLIA)-certified, high-complexity laboratory settings only (87.2%). Less than 10% of the NATs are authorized to be performed in either CLIA-certified, high- or moderate-complexity laboratories (8.9%) while only 3.9% of the NATs can be performed in either CLIA-certified, high- or moderate-complexity laboratories or CLIA-waived patient care settings.

Most of the authorized NATs also detect two or more regions of the SARS-CoV-2 genome and only 32 (17.8%) are single-target NATs. Given that CoVs generally evolve at a rate of 10^−4^ nucleotide substitutions per site per year with mutations being incorporated into the viral genome during every replication cycle [11], the risk of diagnostic drift can be minimized by selecting conserved regions that are relatively stable when a SARS-CoV-2-specific primer-probe set is designed. Overall, the *N* gene is the most commonly targeted gene (66.9%) followed by *Orf1ab* (44.0%), *E* (22.3%), *RdRp* (16.6%), *S* (13.6%), *M* (0.6%) and *Orf8* (0.6%). Although the majority of authorized tests focused on the sequence variations that exist in one or more of these genes to identify SARS-CoV-2, a few RT-PCR tests also utilized the *N* and/or *E* genes for subgenus-specific detection of *Sarbecovirus*. The comparison of all the FDA-EUA NATs are summarized in Table 1 with the complete details available in supplementary Table S1.

## 4. Specimen Collection

Once a decision has been made to pursue SARS-CoV-2 testing in consultation with a healthcare provider, the CDC recommends the collection of an upper respiratory specimen as soon as possible regardless of the time of symptom onset [61]. Nasopharyngeal (NP), oropharyngeal (OP) and nasal specimens collected by swab, aspiration or wash are acceptable for initial diagnostic testing but these specimens must be collected by a trained healthcare provider. The only exception to these is the nasal swab specimen that can be self-collected at home (anterior nares swab) or at a testing site under supervision (nasal mid-turbinate and anterior nares swab). A recent revision made on 8 October 2020 has included saliva as an acceptable specimen that can be self-collected at home or at a testing site under supervision. Although NP swab is no longer the preferred swab-based specimen for SARS-CoV-2 testing as of 29 April 2020 [61], the procedure to obtain it can be unpleasant and more invasive as compared to self-collected nasal swab and saliva that is easier and painless to perform. The inclusion of self-collected nasal swab and saliva as acceptable specimens by the CDC would reduce the reliance on healthcare providers and limit the use of personal protective equipment that are required to perform sampling on-site. Compared to swabs that are relatively cheaper, easier and convenient to use, the collection of aspirates or washes require an additional device in the form of a suction apparatus, making the technique unfeasible for widespread use.

Proper specimen collection is imperative to the success of laboratory diagnosis because the quantity and quality of the specimen can directly affect the accuracy of SARS-CoV-2 NATs. Flocked swab is generally recommended for the collection of most swab-based specimens as the increased surface area of the tip and the ease of particulate matter elution can lead to a greater yield of specimen [62]. More importantly, swabs with calcium alginate tips or wooden shafts should not be used because the possible presence of inhibiting substances can give rise to false negative test result. Based on the CDC guidelines [61], specimens should only be collected using sterile synthetic fiber swabs with plastic or wire shafts and placed immediately into a sterile transport tube containing transport medium or saline unless specified otherwise in the instruction for use (IFU)/EUA summary of the NAT used. Preservative is not required for saliva specimen that is collected in a sterile, leak-proof screw cap container. Specimens that will be processed within 72 h after collection can be stored at 2–8 °C. Otherwise, ultra-low temperature freezer will be a requisite as the specimens are to be stored at −70 °C or below if a delay in testing or shipping is expected.

Testing lower respiratory tract specimens has been deemed optional as most of these specimens have greater technical skill and equipment requirements to collect. The CDC recommends the collection of sputum for patients with a productive cough but other lower respiratory tract specimens such as bronchoalveolar lavage (BAL), tracheal aspirate, pleural fluid and lung biopsy should only be collected when clinically indicated, such as in patients receiving invasive mechanical ventilation. All lower respiratory tract specimens are collected in a sterile, leak-proof, screw-cap collection cup or sterile dry container. As of 31 October 2020, the CDC’s interim guidelines for collecting, handling, and testing clinical specimens from persons for coronavirus disease did not address whole blood, serum or plasma specimens for SARS-CoV-2 diagnostic testing [61].

## 5. Preparation of Specimen for NATs

For most of the FDA-EUA NATs, collected specimens must be processed to ensure that high quality viral RNA template is obtained for subsequent downstream analysis if SARS-CoV-2 is present. During the RNA extraction process, impurities in the specimen matrix that can potentially inhibit downstream analysis are removed before the concentrated viral RNA is eluted. Given that the quality and integrity of the isolated RNA can directly affect the sensitivity of NATs, utmost care and precautions must be taken to prevent the degradation of RNA. Presently, a wide range of commercial RNA extraction kits have been validated for use in different FDA-EUA NATs, but the extraction process generally follows four important steps comprising of cell lysis, protein denaturation, RNA purification and RNA elution by using either silica-based spin column or magnetic particle-based method. Cell lysis is most commonly achieved using chaotropic salts such as guanidine hydrochloride and guanidine thiocyanate, whereas protein contaminants can be removed through enzymatic digestion using proteinase K. The selective binding of RNA to the spin column or magnetic particles allows RNA to be isolated and eluted at the end of the extraction protocol.

### 5.1. Spin Column Method

In the spin column method, the isolation of RNA is based on the affinity binding between negatively charged RNA and positively charged silica membrane in the spin column under alkaline or high salt condition. This allows other cellular components such as protein, carbohydrate and lipid to be washed out while RNA is retained at the silica membrane. To elute RNA from the silica membrane, a hyposmotic solution is used, such as a Tris-EDTA buffer or nuclease-free water. The spin column method is simple and easy to perform but a centrifuge or a vacuum manifold with vacuum source is required to force the solution through the silica membrane. Furthermore, the silica membrane in the spin column can be potentially clogged by a large amount of debris and this can adversely affect the RNA yield and quality. The need for centrifugation or vacuum filtration also limits the extent of miniaturization and automation of the spin column method for high throughput processing. Nonetheless, fully automated, low throughput nucleic acid extraction instruments with integrated centrifuge, heated shaker and liquid handling system have been developed, such as the benchtop QIAcube Connect (Qiagen, Hilden, Germany) that can process up to 12 samples in a single run and even permits remote run monitoring that frees up skilled staff to concentrate on other tasks.

Alternatively, the centrifugation or vacuum filtration could be obviated by placing the silica membrane in a syringe instead of a spin column, such as seen in the Biomeme’s M1 Sample Prep Cartridge Kit [63]. The palm-size, single-use cartridge is not only highly portable but also enables RNA extraction to be performed in resource-limited settings as no power source and additional device are required. However, these advantages will have to be balanced with the increased workload of using the cartridge because only one sample can be processed at a time and the repetitive pumping of the sample through various sections of the cartridge can make it cumbersome to process a large number of samples.

### 5.2. Magnetic Particle-Based Method

Unlike silica membrane, magnetic particles are more efficient at capturing RNA as there is no risk of clogging, but migration of the particles can be impeded in highly viscous samples and residual particles in the eluted RNA can act as a contaminant. Selective binding of RNA under high salt condition is achieved by functionalizing the magnetic particles with silica surfaces, and RNA-bound magnetic particles can be easily separated from the aqueous phase using an external magnetic field. After the removal of unbound substances, RNA can be eluted from the magnetic particles under low salt condition. Large reference laboratories that require high sample processing capability may benefit the most from having a fully automated, high-throughput, magnetic particle-based nucleic acid extraction instruments such as the MagNA Pure 96 Instrument (Roche, Mannheim, Germany) that can process up to 96 samples in less than one hour. Instruments with smaller footprints and lower sample capacities ranging from eight to 48 samples per run are also commercially available from major life science companies including Roche, Qiagen, Promega, and bioMérieux.

### 5.3. Other Specimen Processing Procedures

Although automation of RNA extraction reduces hands-on processing time, increases consistency and minimizes risk of cross-contamination, these instruments are not readily available in most clinical laboratories. Manual RNA extraction using either silica-based spin column or magnetic particle-based method can become tedious due to the amount of liquid handling and transfer that are involved in processing a large number of samples. Therefore, highly simplified RNA extraction protocols that are easy to perform and take less than 15 min to complete have emerged in some of the FDA-EUA NATs. In the Novel Coronavirus (SARS-CoV-2) Fast Nucleic Acid Detection Kit (PCR-Fluorescence Probing) [64], specimen in the SARS-CoV-2 Collection Fluid only requires shaking and mixing for at least one minute before the resulting solution is ready to be added into RT-PCR reaction mixture. Sansure BioTech’s Novel Coronavirus (2019-nCoV) Nucleic Acid Diagnostic Kit (PCR-Fluorescence Probing) [65] also incorporates a simplified extraction protocol that relies on a proprietary RNA fast-releasing technology. Specimen in the Sample Storage Reagent only needs a 5-min centrifugation step followed by 5 min of vortexing to resuspend the pellet in Sample Release Reagent and can be used as a template for RT-PCR reaction.

Other variations of specimen-processing protocols can be observed in Advanta Dx SARS-CoV-2 RT-PCR Assay [66], SalivaDirect [67], Lyra Direct SARS-CoV-2 Assay [68] and Hymon SARS-CoV-2 Test Kit [69]. Both Advanta Dx SARS-CoV-2 RT-PCR Assay and SalivaDirect are designed for testing saliva specimens that are treated with a simple heating step, and while the former uses a non-enzymatic approach (RNAsecure, Thermo Fisher Scientific) to inactivate RNases, the latter uses proteinase K. Likewise, only a 10-min lysis procedure at 95 °C is required for swab specimens eluted into the Process Buffer in the Lyra Direct SARS-CoV-2 Assay. The Hymon SARS-CoV-2 Test Kit provides a master mix containing lysozyme to be added to the specimen and after 2 incubation steps (10 min at 58 °C and 2 min at 95 °C), the lysed specimen can be added into RT-PCR reaction mixture. There are also instances whereby the specimen-processing step is eliminated from the test procedure, such as in the qSanger-COVID-19 Assay [70], LumiraDx SARS-CoV-2 RNA STAR Complete [71], and MobileDetect Bio BCC19 Test Kit [72]. These tests allow specimen-containing transport medium to be added directly into the amplification reaction mixture. The varying degree of complexity in the specimen-processing step of FDA-EUA NATs should be given due consideration as it relates directly to the availability of consumables, equipment, and human resources as well as the expected turnaround time.

## 6. Controls for NATs

Regardless of the technology used, NATs are not foolproof and require controls to ensure that the results obtained are reliable. Since multiple factors that include the use of faulty reagents and equipment, contamination, presence of inhibiting substances in the specimen matrices as well as human and technical errors can cause SARS-CoV-2 NAT results to be invalid, finding the possible cause(s) can be almost impossible without the inclusion of appropriate controls. The process controls, which are typically analyzed with every batch of specimens tested, are used to verify the proper functioning of the molecular diagnostic workflow. In the FDA-EUA NATs, the positive process control (PPC) can be either SARS-CoV-2-negative clinical specimens spiked with SARS-CoV-2 source materials or SARS-CoV-2-positive clinical specimens while SARS-CoV-2-negative clinical specimens and non-infectious, cultured human cell lines are used as negative process control (NPC). After subjecting the PPC and NPC to the entire protocol along with other clinical specimens to be tested, a positive and negative result should be obtained with the PPC and NPC, respectively. The primary purpose of the PPC is to verify that all the steps, starting from specimen processing until amplicon detection, are working as intended. The NPC, which also serves as a negative extraction control, verifies the absence of accidental cross-contamination from other positive samples that are analyzed at the same time. If the expected results were not obtained with the PPC and NPC, the test results obtained for that particular batch will be considered invalid and the specimens will have to undergo the entire test procedure again.

Some of the FDA-EUA NATs require the spiking of clinical specimens to be tested with non-target RNA, such as MS2 phage and armored RNA, that acts as both positive extraction control and exogenous internal control (IC). As the exogenous IC is simultaneously extracted and amplified with the target RNA in the same tube, its detection serves to rule out false-negative result that may arise from the failure of RNA extraction, improper amplification reaction set up, use of faulty reagents or equipment and inhibition of amplification reaction. However, clinical specimens to be tested need not be spiked if an endogenous IC is detected instead, such as the human RNase P and β-actin that are naturally present in human specimens. The other two types of controls that are commonly included in an amplification run are the no template control (NTC) and positive control (PC). In terms of the composition, the NTC and PC contain all the amplification reaction components but the sample in NTC is replaced with nuclease-free water, while in PC it can be either SARS-CoV-2 genomic RNA, in vitro transcribed target RNA or a pseudovirus/plasmid DNA containing the target gene sequence(s). The result of NTC should always be negative because a positive NTC result indicates that cross-contamination has occurred prior to the amplification process. Likewise, a negative result obtained with the PC also invalidates the amplification run as failure of the PC to be amplified and detection indicates that the amplification process is not performing as intended. Adherence to the best practices outlined in the IFU/EUA summary of each FDA-EUA NAT is important to ensure the quality of SARS-CoV-2 testing.

## 7. Real-Time RT-PCR

RNA-based viruses, like SARS-CoV-2, require the PCR technique to be coupled with reverse transcription before the genetic material can be selectively amplified [73]. The reverse transcription process relies on reverse transcriptase to synthesis a DNA:RNA hybrid from the targeted region of a viral RNA genome that is flanked by primers. The RNase H activity of the reverse transcriptase causes the RNA portion of the hybrid to be degraded and a single-stranded complementary DNA (cDNA) copy of the targeted RNA region is produced. DNA polymerase then converts the single-stranded cDNA into double-stranded DNA (dsDNA) that in turn becomes the template for PCR. PCR consists of three thermal cycling steps (denaturation, annealing and extension) and these steps are generally repeated 45 times to amplify the target DNA. The incorporation of a fluorescent dye or probe(s) in the reaction mixture enables the amount of replicated DNA to be measured in real-time through the increase in fluorescent signal. The number of cycles that are needed for the fluorescent signal to exceed the threshold within the exponential phase of the amplification reaction is denoted as the cycle threshold (Ct) [74].

The integration of amplification and detection within a closed-tube system is highly desirable, as it does not only obviate the need for post-amplification analysis but also significantly minimizes the risk of carry-over contamination [75]. Among the various fluorogenic detection systems, TaqMan probes remain the most popular option among FDA-EUA real-time RT-PCR tests. Contrary to the use of a generic DNA intercalating dye, the use of a probe confers sequence-specific detection, permits multiplexing, and minimizes non-specific signal from PCR artifacts. Due to the close proximity between the fluorophore and quencher in a TaqMan probe, no fluorescent signal will be released as long as the oligonucleotide remains intact. When a target sequence is present, the binding of TaqMan probe to the complementary region will result in the hydrolysis of the probe during polymerization due to the 5′-nuclease activity of DNA polymerase. Separated from the quencher, the fluorescence emission by the fluorophore will be restored and this leads to a net increase in the fluorescence detected by the optical system. Because the TaqMan probe has to be annealed to its target during polymerization, a two-step PCR protocol that consolidates the annealing and extension into a single step is used, leading to a shorter run time as compared to that of the conventional three-step PCR protocol. In a multiplex format, different primer-probe sets are added into a single RT-PCR reaction to simultaneously amplify two or more targets. Besides saving cost, time and labor, multiplexing also increases the throughput of real-time RT-PCR platform.

As the first real-time RT-PCR test to be authorized under EUA, the present CDC 2019-nCoV Real-Time RT-PCR Diagnostic Panel targets two different regions of the *N* gene (*N1* and *N2*) for the qualitative detection of SARS-CoV-2 in various types of upper and lower respiratory tracts specimens. RNA extraction is required and it can be performed either manually or with automated nucleic acid extraction systems that have been validated for use with the test. The panel provides the primer-probe sets for the target *N* gene and IC (RNase P), but the RT-PCR reaction has to be set up using a validated commercial enzyme master mix. The PC material (in vitro transcribed RNA) is also provided but not the human specimen control that is required as a NPC. Subsequent real-time RT-PCR amplification and detection will take approximately 1 h and 20 min to complete. When all the controls exhibit the expected performance, a specimen is considered positive for SARS-CoV-2 if both *N1* and *N2* are positive (Ct < 40), whereas if only RNase P is positive (Ct < 40), the specimen is considered negative. If only one of the two targets is positive, an inconclusive result is obtained and the specimen has to be retested. Likewise, the extraction and amplification process have to be repeated for a particular sample when neither RNase P, *N1* nor *N2* is positive. The LoD of the CDC 2019-nCoV Real-Time RT-PCR Diagnostic Panel is in the range of 10^0^–10^0.5^ copies/µL and the test obtained 100% positive percent agreement (PPA; 95% CI: 77.2–100%) and negative percent agreement (NPA; 95% CI: 96.4–100%) with a composite comparator [4].

### 7.1. Multiplexed Detection of SARS-CoV-2 and Other Pathogens

Besides detecting multiple targets of the SARS-CoV-2 genome, the multiplexing capability of the RT-PCR platform has been capitalized for the simultaneous detection of other viruses and/or bacteria. With the arrival of the influenza season, the CDC’s Influenza SARS-CoV-2 (Flu SC2) Multiplex Assay [76], Roche’s cobas SARS-CoV-2 & Influenza A/B [77], and Cepheid’s Xpert Xpress SARS-CoV-2/Flu/RSV [78] not only detect but also differentiate between SARS-CoV-2, Influenza A and Influenza B, with the latter detecting RSV as well. Furthermore, the use of highly multiplexed assays such as the QIAstat-Dx Respiratory SARS-CoV-2 Panel (Qiagen) [79] and BioFire Respiratory Panel 2.1 (BioFire Diagnostics) [80] may be warranted as the co-infection of SARS-CoV-2 with one of more pathogens have been reported in multiple clinical studies [81,82,83,84] especially among severe COVID-19 cases [84,85,86]. Both panels are designed to simultaneously identify 22 bacteria and viruses including rhinovirus/enterovirus, influenza A, influenza B, other CoVs, RSV, parainfluenza, metapneumovirus, *Mycoplasma pneumonia* and *Chlamydophila pneumonia* that have been reported to cause co-infections among COVID-19 patients [87]. However, the feasibility of running these highly multiplexed assays may be limited by the testing capacity of the QIAstat-Dx Analyzer 1.0 Qiagen, Hilden, Germany) (1 sample/hour/analytical module) and BioFire FilmArray 2.0/Torch System (BioFire, Salt Lake City, UT, USA) (1 sample/hour/FilmArray). Nevertheless, these panels can reduce the risk of under-diagnosis, provide a better understanding of the prevalence of COVID-19 co-infection and produce empirical evidence to support development of drug prescription policy for COVID-19 patients.

### 7.2. Automated Sample-to-Result and Point-of-Care Systems

The majority of the real-time RT-PCR tests were validated in 96-well, real-time thermal cyclers but more importantly, plug-and-play systems with complete automation from sample preparation to result interpretation have come as a solution to staff-constrained laboratories facing an increased demand for SARS-CoV-2 testing services. Automating the molecular diagnostic workflow not only reduces the risk of exposure and working time of the laboratory personnel, but also increases reproducibility, accuracy of the result and overall efficiency of the laboratory operations. The suitability of an automated system for SARS-CoV-2 testing in a diagnostic laboratory depends on a number of factors including the instrument-reagent costs, the laboratory space requirement, complexity of the instrument, testing capacity, hands-on time and time-to-result. Generally, fully automated systems with high-throughput capacity tend to involve large, complicated and costly instruments that are meant for a laboratory setting. In order to cater to other healthcare and resource-limited settings, the development of low-complexity, portable and robust systems that need not be operated by professionally trained personnel would be ideal. For instance, the Biomeme’s Franklin Thermocycler is a highly portable device that can be wirelessly paired to a smartphone to enable multiplex real-time detection of SARS-CoV-2 in up to 9 samples with the Biomeme SARS-CoV-2 Real-Time RT-PCR Test [63]. In combination with the M1 Sample Prep Cartridge Kit, which is used for RNA extraction without any laboratory equipment or electricity, the sample-to-result time is only less than 1 h. Despite providing a mobile solution for SARS-CoV-2 testing, the Biomeme SARS-CoV-2 Real-Time RT-PCR Test is presently limited to CLIA-certified, high-complexity laboratories. Nevertheless, there are four real-time RT-PCR tests that are authorized for use at POC settings.

The Cepheid’s Xpert Xpress tests [78,88] and Roche’s cobas SARS-CoV-2 & Influenza A/B Nucleic Acid Test on the cobas Liat System [89] are the epitome of simplicity as the hands-on time is less than one minute, during which an aliquot of specimen-containing viral transport medium (VTM) or saline is transferred into a self-contained cartridge or assay tube before the test is ready to be run. The entire nucleic acid workflow including sample preparation, RNA extraction, RT-PCR amplification, target detection and report generation is fully automated by an integrated, benchtop system. With the cobas Liat System (Roche, Mannheim, Germany), the sample-to-result time of cobas SARS-CoV-2 & Influenza A/B Nucleic Acid Test is only 20 min as compared to the 45-min Xpert Xpress tests. Additionally, the cobas SARS-CoV-2 & Influenza A/B Nucleic Acid Test also showed better performance characteristics in terms of LoD (12 copies/mL), PPA (100%, 95% CI: 93.6–100%) and NPA (100%, 95% CI: 98.4–100%) than Xpert Xpress SARS-CoV-2/Flu/RSV [89]. The Xpert Xpress SARS-CoV-2/Flu/RSV, which is run on the GeneXpert Instrument System (Cepheid, Sunnyvale, CA, USA), has a higher LoD (131 copies/mL) and lower values of PPA (97.8%, 95% CI: 88.4–99.6%) and NPA (95.6%, 95% CI: 85.2–98.8%) [78]. The BioFire’s RP2.1-EZ has a similar run time as that of Cepheid’s Xpert Xpress tests (45 min) but there are more manual steps involved before the pouch can be loaded and run on the fully automated FilmArray 2.0 instrument (BioFire, Salt Lake City, UT, USA). Despite the higher LoD (500 copies/mL), the RP2.1-EZ demonstrated a PPA of 100% (95% CI: 92.9–100%) and a NPA of 100% (95% CI: 72.2–100%) [90].

## 8. End-Point RT-PCR

Unlike real-time RT-PCR that focuses on the exponential phase of the amplification reaction for Ct value determination, end-point RT-PCR relies on the detection of the accumulated product at the end of the amplification reaction where it has usually entered into the plateau phase. In this section, we will discuss the various end-point detection methods that are used in FDA-EUA end-point RT-PCR tests including lateral flow, fluorescence, enzyme-based colorimetric, electrochemical, magnetic resonance and matrix-assisted laser desorption ionization-time of flight (MALDI-TOF).8.1. Lateral Flow Detection

Mesa Biotech’s Accula SARS-CoV-2 Test [91] combines RT-PCR with an immunoassay-based lateral flow device for visual end-point detection and received EUA for use at POC settings. The lateral flow device is integrated within a microfluidic test cassette along with all the necessary reagents for virus lysis and RT-PCR amplification. A major advantage of substituting real-time RT-PCR monitoring with a lateral flow-based end-point detection is the further miniaturization of the analyzer by removing the need for bulky optical system. The 30-min, fully automated, sample-to-answer test uses a palm-sized dock (Accula Dock or Silaris Dock; Mesa Biotech, San Diego, CA, USA) to control the reaction temperatures, timing and fluid movements within the self-contained test cassette. The test procedure is simple as only an aliquot of SARS-CoV-2 Buffer containing solubilized specimen has to be added into the test cassette that has been placed onto the dock. The dock automatically begins the test program upon the closing of the lid and once the program has completed, the test cassette can be removed from the dock for manual interpretation of the lateral flow strip. Although the Accula SARS-CoV-2 Test can be easily performed and requires very minimal operator interaction, the reliability of the assay is dependent on the subjective interpretation of the operator, making the test prone to operator bias. Furthermore, color formation beneath the surface of the membrane (>10 µm) is invisible to the naked eyes as it is masked by the opacity of the membrane [92]. The PPA and NPA of the test established using retrospective clinical specimens were 95.8% (95% CI: 78.9–99.9%) and 100% (95% CI: 86.8–100%), respectively. With a LoD of 200 copies/reaction, the assay has a lower analytical sensitivity as compared to real-time RT-PCR tests developed by GeneMatrix (NeoPlex COVID-19 Detection Kit, 50 copies/reaction) [93] and Access Bio (CareStart COVID-19 MDx RT-PCR, 10 copies/reaction) [94].

### 8.2. Enzyme-Based Colorimetric Detection

The Rheonix COVID-19 MDx Assay [95] uses a proprietary, self-contained CARD cartridge that houses a microfluidic network, pumps, valves and individual assay chamber for four different samples. Up to six CARD cartridges can be loaded into the full automated, benchtop Encompass MDx Workstation (Rheonix, New York, NY, USA) for the simultaneous processing of 24 samples in less than five hours. The detection principle of the Rheonix COVID-19 MDx Assay is based on enzyme-catalyzed formation of colored precipitate. More importantly, the workstation’s software automatically acquires the signal intensity and interprets the results, eliminating the variability associated with subjective visual interpretation. The workstation automatically transfers the samples into the CARD cartridges where virus lysis, RNA extraction, RT-PCR amplification and detection take place. The single-stranded biotinylated amplicons are flowed over an array of capture probes embedded within the CARD cartridge and the resulting hybridization complexes are tagged with streptavidin-conjugated horseradish peroxidase (HRP), allowing colored precipitate to be formed upon the addition of substrate. The Rheonix COVID-19 MDx Assay is limited to CLIA-certified, high-complexity laboratories but the total assay time for 24 samples is shorter and the analytical sensitivity (25 genomic equivalents/reaction) is higher as compared to those of Mesa Biotech’s Accula SARS-CoV-2 Test.

Unlike other molecular diagnostic solutions that require an analyzer or instrument to run the tests, the Visby COVID-19 Test [96] is a palm-sized, self-contained device that runs on its own when powered by electricity. To run the 30-min test, diluted specimen is first loaded into the sample port of the device. The port is then closed followed by three sequential button pushes. Once the device has been plugged in, the test automatically performs the sample preparation, RT-PCR amplification and detection. When present, target amplicons would be anchored via hybridization to specific locations along a flow channel. An enzymatic reaction between HRP and a color producing substrate ensues resulting in an observable color change for a positive reaction. As the enzymatically generated colors in the results window may vary in hue and intensity, a positive signal can be indicated by any shade of color. Given that the Visby COVID-19 Test result is based on visual interpretation, some operator bias is inevitable. PPA and NPA values of the test were found to be 100% (95% CI: 88.6–100%) but the LoD (1112 genomic copies/mL) is almost 3-fold higher than that of real-time RT-PCR test developed by BioMérieux (SARS-COV-2 R-GENE, 380 genomic copies/mL) and BioFire (BioFire COVID-19 Test, 330 genomic copies/mL) [96,97,98].

### 8.3. Fluorescence Detection

The DxTerity SARS-CoV-2 RT-PCR CE Test (DxTerity Diagnostics) [99], Alimetric SARS-CoV-2 RT-PCR Assay (Alimetrix) [100], IntelliPlex SARS-CoV-2 Detection Kit (PlexBio Co. Ltd.) [101], BioCode SARS-CoV-2 Assay (Applied Biocode) [102], and HDPCR SARS-CoV-2 Assay (ChromaCode) [103] rely on an end-point fluorescent detection method but the acceptable specimen(s), working principle, hands-on time and instrumentations of these tests differ from each other.

#### 8.3.1. Capillary Electrophoresis

While various type of respiratory specimens can be tested with the abovementioned NATs, the DxTerity SARS-CoV-2 RT-PCR CE Test only focuses on saliva specimens that are self-collected at home. A total of four monoplex RT-PCR reactions (*N*, *E*, *Orf1ab*, and RNase P) are prepared per sample using different fluorophore-labeled primer mixes (FAM, VIC, NED and Atto-565) for each of the target amplicons. All the amplicons are combined prior to capillary electrophoresis because each of the target amplicon will be separated based on electrophoretic mobility when migrating through the capillary tube under an electric field. Compared to conventional slab gel, capillary electrophoresis is more efficient, faster and provides better resolution [104]. The peak height, which is measured in relative fluorescent unit (RFU), of each target amplicon is normalized to the peak height of an internal size standard for each injection before target-specific thresholds are applied for result interpretation. The target population to be analyzed with DxTerity SARS-CoV-2 RT-PCR CE Test is a factor to be considered given that the PPA and NPA values differed in clinical studies between symptomatic (PPA, 97.3%; NPA, 90%) and asymptomatic (PPA, 84.6%; NPA, 99%) individuals.

#### 8.3.2. Digital Multiplexing Technologies

The IntelliPlex SARS-CoV-2 Detection Kit and BioCode SARS-CoV-2 Assay do not rely on different fluorophores to detect multiple targets of the SARS-CoV-2 genome but employ a digital multiplexing strategy known as πCode MicroDisc and barcoded magnetic beads (BMB) technologies, respectively. Each of the πCode MicroDisc with a distinct image pattern and the BMB barcode corresponds to a specific capture probe and the pooling of different πCode MicroDiscs or BMB enables multiple target amplicons to be detected in a single well. Hybridization complexes, which consist of single-stranded biotinylated amplicons and capture probes conjugated to the surface of the disc or bead, are tagged with streptavidin-phycoerythrin (SAPE) so that the optical imaging system can decode the discs or beads under bright-field and measure the fluorescent signal intensity under dark-field. Despite the similarity in the working principle and turnaround time (~5 h), there are more specialized equipment and manual steps involved in the IntelliPlex SARS-CoV-2 Detection Kit as compared to those of BioCode SARS-CoV-2 Assay. In the Alimetrix SARS-CoV-2 RT-PCR Assay, multiple biotinylated target amplicons (*Orf1ab*, *N1*, *N2*, RNase P and MS2 phage) are also generated but a microarray format is used for detection instead. Each microarray well is spotted with specific probes to detect the targets of interest and upon hybridization with the complementary biotinylated amplicons, the addition of SAPE allows the hybridization complexes to be detected and imaged with a fluorescence array scanner. The fluorescence intensity levels are measured with a custom-built software and used for result interpretation.

#### 8.3.3. HDPCR Technology

The HDPCR SARS-CoV-2 Assay is a particularly attractive fluorescence-based, end-point detection test because it uses a cloud-based software (ChromaCode Cloud) for post-amplification data analysis instead of a physical instrument. Although the multiplexing capability of HDPCR technology is not as extensive as those of πCode MicroDisc and BMB technologies, the uniqueness lies in its ability to expand the multiplexing capability of conventional real-time systems up to four times without changes to the workflow or to the instrument hardware or software. In the HDPCR SARS-CoV-2 Assay, the TaqMan probes are present at a reaction limiting concentration and five ChromaCode Calibrators are included in each RT-PCR run to scale and compare sample data as well as to normalize the expected values across different real-time systems. Considering that the amplification data has to be exported from the real-time system and uploaded onto ChromaCode Cloud for interpretation, internet access is a requisite and the network infrastructure must be reliable and capable of supporting the data transfer. Similar to Alimetrix SARS-CoV-2 RT-PCR Assay, a 4-fold difference in the test LoD (250 copies/mL versus 1000 copies/mL) highlights the importance of extraction method and instrument selection in achieving the lower LoD of the test. Nevertheless, higher analytical sensitivities were reported for the IntelliPlex SARS-CoV-2 Detection Kit (140 copies/mL) and DxTerity SARS-CoV-2 RT-PCR CE Test (50 copies/mL). In addition to the LoD, the same factors also influence the PPA and NPA values of Alimetrix SARS-CoV-2 RT-PCR Assay (PPA, 97.2–100%; NPA, 95.2%) and HDPCR SARS-CoV-2 Assay (PPA, 100%; NPA, 96.7–100%).

### 8.4. Electrochemical Detection

GenMark Diagnostic developed the ePlex SARS-CoV-2 Test [105] and ePlex Respiratory Pathogen Panel 2 (ePlex RP2 Panel) [106] based on the eSensor technology. The hands-on time of both tests are less than 2 min as once the specimen-containing VTM has been loaded into the self-contained ePlex cartridge, the entire nucleic acid testing process starting from extraction to report generation will be automated by the benchtop ePlex instrument (GenMark Diagnostic, Carlsbad, CA, USA). Unlike other hybridization-based assays, exonuclease digestion is used to create single-stranded amplicons followed by competitive DNA hybridization on the surface of gold electrodes arrayed upon printed circuit board (PCB) within the ePlex cartridge. Two probes are utilized per target DNA and formation of the complete hybridization complex allows specific electrical signals generated by the ferrocene-labeled signal probes to be measured via voltammetry. While ePlex SARS-CoV-2 Test only detects 1 viral target, the ePlex RP2 Panel simultaneously detects and identifies 16 respiratory viral targets and two bacterial targets. The modular design of the ePlex system allows its testing capacity to be expanded up to 24 test bays. The sample-to-result time for both tests are under 2 h but ePlex RP2 Panel has better performance characteristics including lower LoD (250 genomic copies/mL) and higher PPA (100%, 95% CI: 93.9–100%) and NPA (100%, 95% CI: 96.7–100%) values as compared to those of ePlex SARS-CoV-2 Test. The LoD, PPA and NPA values of ePlex SARS-CoV-2 Test are 750 genomic copies/mL, 94.4% (95% CI: 74.2–99%) and 100% (95% CI: 92.4–100%), respectively. The analytical sensitivity of ePlex RP2 Panel is also higher than those of several RT-PCR-based assays such as Visby Medical COVID-19 (1112 genomic copies/mL), SARS-COV-2 R-GENE (380 genomic copies/mL) and BioFire COVID-19 Test (330 genomic copies/mL).

### 8.5. MALDI-TOF Detection

The Ethos Laboratories SARS-CoV-2 MALDI-TOF Assay [107] and SARS-CoV-2 MassArray Test [108] are performed in different CLIA-certified, high-complexity laboratories but follow a similar workflow that includes the use of Agena SARS-CoV-2 Panel [109] for multiplexed RT-PCR amplification (*N1*, *N2*, *N3*, *Orf1* and *Orf1ab*) and a MALDI-TOF mass spectrometer for detection of target amplicons. The amplicons have to be subjected to a shrimp alkaline phosphatase treatment to dephosphorylate any unincorporated dNTPs before a single base extension reaction is performed to produce allele-specific extension products with mass-modified dideoxynucleotide terminators. The MassARRAY system (Agena Bioscience, San Diego, CA, USA) is then used to automate all the subsequent processes from sample desalting to data acquisition. Briefly, the desalted extension products are deposited on a silicon chip pre-spotted with matrix crystal before the chip is bombarded by laser irradiation to induce the desorption and ionization of the analyte/matrix co-crystals. Positively charged molecules that accelerate into a vacuum flight tube towards a detector are distinguished by their time-of-flight. The integrated software will review the mass spectrum to identify the targets for result interpretation. The time from RT-PCR amplification to MassARRAY result generation is 8.3 h with 28 min of hands-on time. Due to the variations in RNA extraction kits and instruments used, different LoDs were reported for the Ethos Laboratories SARS-CoV-2 MALDI-TOF Assay (1 TCID_50_/mL), SARS-CoV-2 MassArray Test (0.69–2.75 copies/μL) and Agena SARS-CoV-2 Panel (2.5 copies/μL) with PPA and NPA values of the three tests ranging from 95% to 100%.

## 9. qSTAR

The qSTAR employed in the LumiraDx SARS-CoV-2 RNA STAR [110] and LumiraDx SARS-CoV-2 RNA STAR Complete [71] uses a 2-step cycling protocol that spans only 12 min with the activity of the polymerase and nicking enzyme being relatively favored at the upper (61 °C) and lower (54 °C) temperatures, respectively. During amplification, primers are used to incorporate a nicking site at each end of the duplex amplicon. After the nicking enzyme causes a single-stranded nick in the duplex, the polymerase displaces the downstream non-template strand while it extends the nicked primer to synthesize a new strand, recreating the nicking site in the process. Shuttling between the temperatures leads to multiples copies of the target amplicons being created. Molecular beacons with different fluorophores (FAM and ROX) allow real-time, multiplex detection of the *Orf1a* and IC amplicons. The tests do not use Ct cut-off in the testing algorithm but provide the expected Ct value in accordance to the real-time system used. Unlike LumiraDx SARS-CoV-2 RNA STAR, the LumiraDx SARS-CoV-2 RNA STAR Complete removes the extraction step by allowing specimen-containing VTM to be added directly into the qSTAR reaction mixture containing a proprietary extraction buffer. Hence, the hands-on and total assay time is reduced by combining the lysis and amplification into a single step, but the LoD of LumiraDx SARS-CoV-2 RNA STAR Complete (7500 copies/mL) is 15-fold higher than that of LumiraDx SARS-CoV-2 RNA STAR (500 copies/mL). The PPA and NPA remained comparable between the two tests.

## 10. RT-Digital PCR

The Gnomegen COVID-19 RT-Digital PCR Detection Kit [111] consists of primers and TaqMan probes (*N1*, *N2* and RNase P) that are designed to be used in a single microwell chip, wherein the nucleic acid sample and RT-PCR mixture will be partitioned into as many as 20,000 independent reaction wells for real-time RT-Digital PCR testing. Generally, the assay requires multiple manual steps including RNA extraction, RT-PCR reaction set up and chip preparation. After the amplification process has completed, the chip is imaged and analyzed using QuantStudio 3D Digital PCR System (Thermo Fisher Scientific, Waltham, MA, USA). If the Gnomegen Real-Time Digital PCR Instrument (Gnomegen, San Diego, CA, USA) is used, fluorescence intensity will be measured in real-time as opposed to end-point, as is the case with the QuantStudio 3D Digital PCR System. The test results are interpreted manually and the threshold for negative/positive determination of fluorescent signals are set based on the controls that are included in the RT-PCR run. The LoD was determined to be 8 copies/reaction with 100% PPA and NPA (95% CI: 88.7–100%).

Instead of microwell chip, the Bio-Rad SARS-CoV-2 ddPCR Kit [112] and FastPlex Triplex SARS-CoV-2 detection kit (RT-Digital PCR) [113] rely on a droplet generator to fractionate the RT-PCR reactions into thousands of aqueous droplets within an emulsion oil. Both of the partition-based, end-point RT-PCR tests shared similar workflows consisting of the following steps: RNA extraction, RT-PCR reaction preparation and droplet generation, RT-PCR amplification, droplet reading and data analysis. The Bio-Rad SARS-CoV-2 ddPCR Kit uses QX200 or QXDx AutoDG ddPCR System (Bio-Rad, Hercules, CA, USA) for droplet generation (up to 20,000 droplets) and fluorescence intensity measurement whereas the FastPlex Triplex SARS-CoV-2 detection kit (RT-Digital PCR) uses DropX-2000 Digital PCR System (RainSure Scientific, Suzhou, China) for droplet generation (up to 25,000 droplets), amplification and measurement of droplets diameter, count and fluorescence intensity for result interpretation. Although the LoD of Bio-Rad SARS-CoV-2 ddPCR Kit (150 copies/mL) is lower than that of FastPlex Triplex SARS-CoV-2 detection kit (RT-Digital PCR) (571.4 copies/mL), the PPA and NPA values of FastPlex Triplex SARS-CoV-2 detection kit (RT-Digital PCR) are higher than those of Bio-Rad SARS-CoV-2 ddPCR Kit.

## 11. Isothermal Nucleic Acid Amplification

Isothermal nucleic acid amplification protocols do not involve thermal cycling as these techniques are carried out at a constant temperature. Isothermal amplification-based tests coupled with a RNA extraction-free method have the potential to generate ultra-rapid results, such as those seen in the nicking enzyme amplification reaction (NEAR)-based test, ID NOW COVID-19 (Abbot Diagnostics) [114]. The ID NOW COVID-19 has a sample-to-result time of less than 13 min, nearly twice as fast as the cobas SARS-CoV-2 & Influenza A/B Nucleic Acid Test on the cobas Liat System (20 min) [89]. Another isothermal nucleic acid amplification-based test that received EUA for use under POC settings is the 25-min Cue COVID-19 Test [115]. The assay comprises a Cue Sample Wand for nasal specimen collection and a self-contained Cue cartridge that is designed to be used with a palm-sized, battery-operated Cue Cartridge Reader. The Cue Cartridge Reader is paired to a smart mobile device via Bluetooth in order to transfer the data to the Cue Health App for result interpretation. To perform the assay, the Cue cartridge is first placed into the Cue Cartridge Reader, which heats up the cartridge, before the Cue Sample Wand containing nasal specimen is inserted into the cartridge. Next, the Cue Cartridge Reader automatically activates the Cue cartridge where heating, mixing, amplification, and detection take place.

During isothermal amplification, the target *N* gene and RNase P (IC) are amplified using biotinylated and digoxigenin-labeled forward primers, respectively. The reverse primers for both target and IC are conjugated to HRP. As the target and IC amplicons harbored different labels, the amplicons can be separated using high affinity biotin- and digoxigenin-binding proteins, respectively. Localization of the HRP over a sensing electrode enables the electrical signal generated by the chemical reaction between HRP and its substrate to be detected and converted into a positive or negative results based on a pre-determined cut-off value. The detection rate of Cue COVID-19 Test (75%, 15/20) was reported to be higher than that of the CDC 2019-Novel Coronavirus (2019-nCoV) Real-Time RT-PCR Diagnostic Panel (35%, 7/20) when tested at 1 × LoD of the Cue COVID-19 Test (1.3 genomic copies/µL). However, the LoD of Cue COVID-19 Test remains higher than those of authorized RT-PCR-based POC tests such as the Xpert Xpress SARS-CoV-2/Flu/RSV (131 copies/mL) and cobas SARS-CoV-2 & Influenza A/B Nucleic Acid Test for use on the cobas Liat System (12 copies/mL). Nevertheless, the palm-sized, battery-operated Cue Cartridge Reader makes the Cue COVID-19 Test a highly portable POC test as compared to RT-PCR-based tests that require a benchtop analyzer.

### 11.1. RT-Loop-Mediated Isothermal Amplification (LAMP)

LAMP has been used for the detection of various respiratory viruses including influenza virus [116], RSV [117], human metapneumovirus [118] and other coronaviruses such as NL63 [119] and MERS-CoV [120], but unlike PCR, high amplification efficiency is achieved under isothermal conditions and occurs through DNA polymerase-mediated strand-displacement synthesis. The high specificity of a typical LAMP reaction is conferred by the use of four primers (pairs of outer and inner primers) targeting six distinct regions of the target sequence [121]. LAMP reaction can also be accelerated by including an additional set of loop primers that binds to stem-loops that are not bound by the inner primers. The LAMP amplicons can be monitored in real-time using fluorescent dye/probe(s) or visualized by the naked eye in the form of turbidity or color change (with pH-sensitive/fluorescent dye) at end-point [122]. With the inclusion of a reverse-transcription process, a reverse-transcription LAMP (RT-LAMP) test can be developed for the detection of SARS-CoV-2 RNA.

#### 11.1.1. Fluorescence Detection

The AQ-TOP COVID-19 Rapid Detection Kit [123] and AQ-TOP COVID-19 Rapid Detection Kit PLUS [124] developed by Seasun Biomaterials are multiplex RT-LAMP tests. Dual-labeled peptide nucleic acid probes and a real-time system are used in both tests but the AQ-TOP COVID-19 Rapid Detection Kit PLUS requires two separate reactions for the detection of two target genes (*Orf1ab* and *N* genes) whereas AQ-TOP COVID-19 Rapid Detection Kit only detects the *N* gene in a single reaction. In both tests, the target gene will be co-amplified with RNase P (IC). During amplification at 60°C, the incorporation of fluorescence resonance energy transfer (FRET) probes in the amplicons leads to an increase in the fluorescent signal. A positive amplification reaction is denoted by a Ct value of ≤30. Although the PPA and NPA values of both tests are 100%, the LoD of AQ-TOP COVID-19 Rapid Detection Kit PLUS (1 copy/µL) is slightly lower than that of AQ-TOP COVID-19 Rapid Detection Kit (7 copies/µL) and comparable to the CDC 2019-Novel Coronavirus (2019-nCoV) Real-Time RT-PCR Diagnostic Panel (1–3.16 copies/µL). Due to the shorter amplification duration (~30 min), both RT-LAMP tests can be completed in less than half the expected time of the CDC 2019-Novel Coronavirus (2019-nCoV) Real-Time RT-PCR Diagnostic Panel.

The Pro-AmpRT SARS-CoV-2 Test [125] is a monoplex, closed-tube RT-LAMP test that also relies on a real-time system to measure fluorescence intensity during the amplification of the target *Orf1ab* gene. Although the run time for RT-LAMP is similar to those of Seasun Biomaterials’ AQ-TOP tests, the result interpretation of Pro-AmpRT SARS-CoV-2 Test is not based on Ct value but a reaction time cut-off and the melt profile of the amplicon. The Pro-AmpRT SARS-CoV-2 Test does not have an IC and uses a fluorescent dsDNA intercalating dye that does not confer sequence-specific detection but allow a melt curve analysis to be performed at the end of the amplification reaction. The primary purpose of the melt curve analysis is to identify the presence of non-specific amplicons with different thermal profiles. A positive amplification reaction is denoted by a minimum fluorescence of 10,000 RFU within 24 min and an amplicon melting temperature of 84 ± 1 °C. The LoD of the test was determined to be 125 genomic equivalents/swab with a PPA of 96.6% (95% CI: 83.3–99.4%) and NPA of 100% (95% CI: 88.7–100%).

#### 11.1.2. Colorimetric Detection

Color Genomics does away with the need for a real-time system by developing a RT-LAMP test that uses a microplate reader for amplification and detection of amplicons. In addition to the microplate reader, the Color Genomics SARS-CoV-2 RT-LAMP Diagnostic Assay [126] is performed using two automated systems for RNA extraction and RT-LAMP reaction set up, respectively. For each sample, three different monoplex RT-LAMP reactions are required to amplify the target genes (*N* and *E* genes) and IC (RNase P). During the amplification process, which is carried out at 65 °C in the microplate reader, the incorporation of dNTPs into nascent DNA releases by-products that include hydrogen ions. The accumulation of hydrogen ions causes the pH to drop and consequently, the pH-sensitive dye (phenol red) in the RT-LAMP reaction mixture changes from pink to yellow. The color change is measured spectrophotometrically once per minute over a period of 70 min and a gain in the absorbance ratio (A_430_/A_560_) of ≥0.25 from baseline to end-point is denoted as a positive amplification reaction.

Likewise, Detectachem’s MobileDetect Bio BCC19 Test Kit [72] also uses a pH-sensitive dye for the detection of RT-LAMP amplicons but relies on visual interpretation of color change. Positive and negative amplification reactions are indicated by a yellow and red color, respectively, whereas other hues of colors are considered invalid. The multiplex test does not differentiate between the two target genes (*N* and *E* genes) and an IC is also not included in the test. The sample-to-result time is shorter than that of Color Genomics SARS-CoV-2 RT-LAMP Diagnostic Assay because no RNA extraction is required and the amplification process at 65 °C only takes 30 min. However, lower analytical sensitivity is attained as the LoD of MobileDetect Bio BCC19 Test Kit (75 copies/μL) is 100-fold higher than that of Color Genomics SARS-CoV-2 RT-LAMP Diagnostic Assay (0.75 copies/μL). Color Genomics SARS-CoV-2 RT-LAMP Diagnostic Assay also has 100% PPA (95% CI: 90.6–100%) and 100% NPA (95% CI: 99.2–100%) while MobileDetect Bio BCC19 Test Kit has 97.7% PPA (95% CI: 88–100%) and 100% NPA (95% CI: 94.3–100%).

#### 11.1.3. Clustered Regularly Interspaced Short Palindromic Repeats (CRISPR)-Based Detection

The Sherlock CRISPR SARS-CoV-2 kit [127], SARS-CoV-2 RNA DETECTR Assay [128] and SARS-CoV-2 DETECTR Reagent Kit [129] are the only CRISPR-based, RT-LAMP tests that are authorized under EUA. The Sherlock CRISPR SARS-CoV-2 kit is designed to detect two target genes (*Orf1ab* and *N* genes) and an IC (RNase P) in the monoplex format. As the RT-LAMP amplicons must be transcribed in order to activate the collateral cleavage activity of the programmed CRISPR complex, the T7 polymerase promoter is introduced into the amplicons during a 40-min incubation at 61 °C. The CRISPR-Cas reaction is then carried out in a fluorescence microplate reader at 37 °C to allow transcription to take place. Collateral activation of Cas13 by the transcribed target RNA leads to the cleavage of reporter molecules and a corresponding increase in the fluorescent signal. A positive reaction is denoted by a minimum of 5-fold increase in fluorescence measurement over the corresponding NTC at minute 10. The test demonstrated 100% PPA (95% CI: 83.9–100%) and 100% NPA (95% CI: 88.6–100%) [127].

The DNA endonuclease-targeted CRISPR trans reporter (DETECTR)-based tests (SARS-CoV-2 RNA DETECTR Assay and SARS-CoV-2 DETECTR Reagent Kit) are based on a different RNA-guided RNase (Cas12) but the collateral activity, which is activated upon recognition of its target, is similarly used to cleave bystander reporter molecules. More importantly, the use of Cas12 obviates a post-amplification, transcription step because Cas12 recognizes dsDNA as the activator whereas Cas13 recognizes single-stranded RNA as the activator [130]. Both of the DETECTR-based tests require a real-time system to detect the target *N* gene and RNase P in the monoplex format. The RT-LAMP reaction is first carried out at 62 °C for 30 min followed by the trans-cleavage assay at 37 °C, during which Cas12-mediated cleavage of reporter molecules results in an increase in fluorescent signal. A cut-off value of 500,000 RFU is used to interpret positive/negative result for the target and control. SARS-CoV-2 RNA DETECTR Assay and SARS-CoV-2 DETECTR Reagent Kit shares the same PPA value of 95% (95% CI: 83.5–98.6%) and NPA value of 100% (95% CI: 94.2–100.0%) [128,129].

Overall, the LoD of Sherlock CRISPR SARS-CoV-2 kit (6.75 copies/µL) is lower than those of SARS-CoV-2 RNA DETECTR Assay and SARS-CoV-2 DETECTR Reagent Kit (20 copies/µL) but higher than that of the CDC 2019-Novel Coronavirus (2019-nCoV) Real-Time RT-PCR Diagnostic Panel (1–3.16 copies/µL). There are also more manual steps involved in these CRISPR-based, RT-LAMP tests as compared to conventional multiplex, real-time RT-PCR tests because the monoplex format is used and a post-amplification, cleavage assay is required. The preparation of CRISPR-Cas master mixes from 8 reaction components also increases the hands-on time of Sherlock CRISPR SARS-CoV-2 kit whereas pre-prepared DETECTR master mixes are provided in the SARS-CoV-2 DETECTR Reagent Kit. The open-tube format of the CRISPR-based, RT-LAMP tests also poses a high risk of aerosol contamination as RT-LAMP reaction can yield a large amount of amplicons due to its higher amplification efficiency [131]. Hence, utmost care must to be taken to prevent carry-over and cross-contamination during pre- and post-amplification activities.

### 11.2. NEAR

NEAR is an isothermal amplification technology that is powered by the synergistic action of a nicking enzyme and a strand-displacing DNA polymerase. The repeated nicking, polymerization and strand displacement that can occur at a single restriction site meant that thousands of amplicon can be generated from one primer, conferring NEAR with a high amplification efficiency [132]. The ID NOW COVID-19 (Abbot Diagnostics) [114], which uses the NEAR technology to generate ultra-rapid result in 13 min, holds the fastest COVID-19 NAAT turnaround time to date and is authorized for use under POC settings. The test comprises a Sample Receiver containing elution/lysis buffer, a Test Base holding two reaction tubes of lyophilized amplification reagents including molecular beacons for the target *RdRp* gene and IC, and a Transfer Cartridge for transferring the eluted sample from the sample receiver to the test base. After the Sample Receiver and Test Base have been inserted into the ID NOW instrument, the swab specimen can be added to the Sample Receiver and an aliquot is then transferred to the Test Base using the Transfer Cartridge. Closing of the instrument lid will start the test whereby heating, agitation, fluorescence detection and result interpretation will be performed automatically. The test result will be displayed as either negative, positive or invalid upon the completion of the test. The test has a LoD of 125 genomic equivalent/mL that is 100-fold lower than that of the real-time RT-PCR-based, Lyra Direct SARS-CoV-2 Assay (1.28 × 10^4^ genomic equivalent/mL) that uses a simple heat step for the direct detection of swab specimens [68,114]. The ID NOW COVID-19 demonstrated 100% PPA at 2 × LoD (95% CI: 83.9–100%) and 5 × LoD (95% CI: 72.3–100%) as well as 100% NPA (95% CI: 88.7–100%).

### 11.3. OMEGA Amplification

The iAMP COVID-19 Detection Kit (Atila Biosystems) [133] is a real-time, closed-tube, isothermal amplification test based on the OMEGA amplification technology. A distinctive feature of OMEGA primers in relation to LAMP primers is the introduction of artificial sequences at the 5′ terminus of one of the inner primers [134]. Hence, the artificial sequence introduced during the amplification process extrudes out when the amplicon folds back on itself. The test is relatively easy to perform as swab specimens only require a 15-min incubation in a sample buffer mix at room temperature before it is ready to be added into the amplification reaction mixture. The test is then carried out in a real-time system at 61 °C for 50 min in order to simultaneously reverse transcribe, amplify and detect two target genes (*Orf1ab* and *N* genes) and an IC (*GAPDH*). Fluorescence readings are taken at 1-min intervals, during which signals generated by the incorporation of FRET probes in the target and IC are detected in the FAM and HEX channel, respectively. A positive amplification reaction is denoted by a Ct value of less than 50. This test can be completed in less than 1.5 h but the LoD (10 copies/µL) is 10-fold higher than that of the multiplex, real-time RT-LAMP-based AQ-TOP COVID-19 Rapid Detection Kit PLUS (1 copies/µL) [124,133]. Clinical evaluation of iAMP COVID-19 Detection Kit at two different sites generated PPA and NPA values that ranged from 96.3–100% and 97–100%, respectively.

### 11.4. TMA

Hologic’s Aptima SARS-CoV-2 Assay [135] relies on transcription-mediated amplification (TMA) which is modelled on the replication strategy of retroviruses and requires the use of reverse transcriptase and T7 RNA polymerase for the direct amplification of RNA [136]. The Aptima SARS-CoV-2 Assay is also incorporated into the workflow of several authorized NATs including Poplar SARS-CoV-2 TMA Pooling assay [137], Quest Diagnostics HA SARS-CoV-2 Assay [138], and LetsGetChecked Coronavirus (COVID-19) Test [139]. The TMA-based test, which detects two regions of the *Orf1ab* gene and an IC, is designed to be performed on the Panther system (Hologic, San Diego, CA, USA) that will automate the RNA target capture, TMA and chemiluminescent-based detection. Collected specimens are first transferred to tubes containing lysis buffer before they are loaded into the Panther system so that the released RNA can bind to target-specific probes containing a poly-A tail. The hybridization complexes are then captured using poly-T probes conjugated to magnetic microparticles. The captured RNA serve as templates for the TMA process and the resulting RNA amplicons are detected via hybridization with probes that are labeled with different acridinium ester molecules. The hybridization complexes are differentiated based on measurements of photon output during the detection read time. Results are interpreted using a cut-off based on the total photon emission (relative light units) and the kinetic curve type. The LoD of Aptima SARS-CoV-2 Assay (83 copies/mL) is generally lower than most of the FDA authorized isothermal and non-isothermal NAATs but remains higher than some of the RT-PCR tests including Wantai SARS-CoV-2 RT-PCR Kit (50 copies/mL), Diagnovital SARS-CoV-2 Real-Time PCR Kit (38 copies/mL) and cobas SARS-CoV-2 & Influenza A/B Nucleic Acid Test for use on the cobas Liat System (12 copies/mL) [89,135,140,141]. The PPA and NPA values were found to be 100% (95% CI: 92.9–100%) and 98.2% (95% CI: 90.4–99.7%), respectively.

## 12. Sanger Sequencing

In addition to the aforementioned detection platforms, sequence-specific detection of SARS-CoV-2 amplicons can also be achieved through sequencing whereby the exact sequence of nucleotides in the amplicon’s fragments are determined. As its name implied, the qSanger-COVID-19 Assay (BillionToOne) [70] is a Sanger sequencing-based assay that requires a thermal cycler for RT-PCR amplification, PCR clean-up and cycle sequencing followed by sequence determination using a Sanger sequencing instrument. Although no specimen-processing step is needed, the protocol remains highly manual as it requires the set-up of multiple reactions. During the amplification process, the target SARS-CoV-2 sequence (*N* gene) is co-amplified with a frame-shifted, spike-in control using the same set of primer pair. The spike-in sequence is identical to the SARS-CoV-2 target sequence except for a 4-bp deletion. The resulting frameshift enables the differentiation of the target SARS-CoV-2 sequence from the spike-in sequence when the electropherogram is analyzed. Following RT-PCR amplification, excess primers and nucleotides are enzymatically removed before cycle sequencing can be performed. After unincorporated dye terminators have been removed from the sequencing reactions, automated Sanger sequencing is performed by capillary electrophoresis. Finally, post-sequencing analysis may present as a challenge given that the electropherograms have to be manually inspected by trained personnel in order to identify the SARS-CoV-2 and/or spike-in sequence alignments. The LoD of qSanger-COVID-19 Assay was determined to be 3200 copies/mL with a PPA value of 90% (95% CI: 74.4–96.5%) and NPA value of 100% (95% CI: 88.7–100%).

## 13. Next Generation Sequencing (NGS)

Unlike Sanger sequencing that is designed to generate a consensus sequence for single-target amplicons, NGS-based technologies allows millions to billions of DNA strands to be sequenced in parallel during a single run [142]. There are five targeted NGS-based tests that are authorized under EUA: four of these tests utilize the Illumina sequencing by synthesis (SBS) technology while Oxford nanopore sequencing technology is employed in the remaining test.

### 13.1. Illumina Sequencing by Synthesis (SBS) Technology

The Illumina COVIDSeq Test [143], which uses the SBS technology with reversible termination, was developed based on a modified version of the ARTIC Network multiplex PCR protocol [144]. The single-read sequencing of 98 target amplicons, which are designed to tile across nearly the entire SARS-CoV-2 genome, and 11 human mRNA (IC) are to be performed on an Illumina sequencing platform (NovaSeq 6000 or NextSeq 500/550/550Dx, Illumina, San Diego, CA, USA). However, the test can be technically complex and labor-intensive due to the high number of manual steps involved in the targeted amplicon sequencing workflow. Firstly, RNA has to be extracted from the specimen and annealed using random hexamers to prepare for cDNA synthesis. After the viral genome has been amplified using two separate PCR reactions, the amplified fragments are then pooled and subjected to tagmentation. The post-tagmentation yield has to be normalized before the adapter-tagged amplicons can undergo a second round of PCR amplification to generate indexed libraries. Once the libraries have been pooled, cleaned up and quantified, they are finally ready to be loaded into the sequencer that will automate the sequencing process.

SBS begins with the clustering of pooled libraries onto a flow cell. During each sequencing cycle, only a single dNTP with its distinct fluorophore will be added to the nucleic acid chain because the nucleotides are modified with an inactive 3′-hydroxyl group that acts as a reversible terminator to polymerization. The fluorescent emission is then imaged to identify the base before the terminator and fluorophore are cleaved to enable the incorporation of another terminator-bound dNTP. A sample is interpreted as positive for SARS-CoV-2 when at least 90 out of the 98 target amplicons are detected. The Illumina COVIDSeq Test is highly scalable as the NovaSeq 6000 system and NextSeq 500/550/550Dx can process up to 3072 and 384 results, respectively, in 12 h. The analytical sensitivity of the Illumina COVIDSeq Test is dependent on the extraction method used as the LoD attained with the Quick-DNA/RNA Viral MagBead Kit (Zymo Research, Irvine, CA, USA; 500 copies/mL) is 2-fold lower than that obtained with QIAamp Viral RNA Mini Kit (Qiagen, Hilden, Germany; 1000 copies/mL). The PPA and NPA values are also influenced by the extraction methods and ranged from 98.1–100% and 97.3–97.4%, respectively [143].

The Guardant-19 [145], UCLA SwabSeq COVID-19 Diagnostic Platform [146] and Helix COVID-19 NGS Test [147] are also targeted amplicon sequencing tests based on the Illumina SBS technology but the testing is limited to different CLIA-certified, high-complexity laboratories. Unlike the Illumina COVIDSeq Test, Guardant-19 only detects a target gene (*N1*) and an IC (RNase P). The extracted RNA from each specimen is subjected to RT-PCR in triplicates, during which plate- and well-specific barcodes will be introduced to uniquely label each sample within a plate of 96 unique samples. The RT-PCR amplification products are then pooled in sets of 96 prior to another PCR run for combinatorial sample barcoding and to enable NGS clustering. After a second round of pooling in sets of 96, the pools are ready for paired-end sequencing on the Illumina NextSeq 500/550 instrument (Illumina, San Diego, CA, USA). Following quality control assessment that includes the evaluation of positive and negative controls as well as post-run quality metrics, a Guardant-19 score is calculated per sample based on the median value of at least two replicate-level score (ratio of SARS-CoV-2 reads to spike-in reads). A cut-off Guardant-19 score of 0.01 is used for SARS-CoV-2 positive/negative interpretation. The LoD of Guardant-19 (125 copies/mL) is 4- to 8-fold lower than that of the Illumina COVIDSeq Test (500–1000 copies/mL) [143,145]. Guardant-19 has a PPA value of 95.5% (95% CI: 87.5–99.1%) and a NPA value of 98.3% (95% CI: 91.1–100%).

Both UCLA SwabSeq COVID-19 Diagnostic Platform and Helix COVID-19 NGS Test simultaneously detects a target gene (*S* gene), a spike-in control and an IC (*RPP30*). The spike-in control differs from the target *S* gene by a 6-nucleotide stretch of sequence and both are amplified using the same set of primer pair. The tests begin with RNA extraction followed by cDNA synthesis with two sets of indexed primers to generate barcoded amplicons and to enable NGS clustering. The resulting barcoded amplicons are pooled, purified and quantified before single-read sequencing is performed on an Illumina sequencing platform. In UCLA SwabSeq COVID-19 Diagnostic Platform, primary analysis is performed using the Real Time Analysis (RTA) software (Illumina, San Diego, CA, USA) and the BCL file obtained is converted into FASTQ sequencing files with bcl2fastq software (Illumina, San Diego, CA, USA). The total number of sequence matched to each amplicon in each sample is counted and these counts are utilized in a decision tree for interpretation of results. Similarly, Helix COVID-19 NGS Test uses the bcl2fastq software to generate the sequencing files which are then fed into the Helix COVID-19 NGS Test Bioinformatics Pipeline. The total number of sequence matched to each amplicon in each sample is counted and after quality control filters were applied, the software will interpret the results using a rule-based algorithm. The LoD of Helix COVID-19 NGS Test (125 genomic copy equivalent/mL) is 2-fold lower than that of UCLA SwabSeq COVID-19 Diagnostic Platform (250 genomic copy equivalent/mL) although both tests demonstrated 100% PPA and NPA [146,147].

### 13.2. Oxford Nanopore Sequencing Technology

The Clear Dx SARS-CoV-2 Test (Clear Labs) [148] is the only authorized targeted NGS-based assay that uses the Oxford nanopore sequencing technology. In contrast to the short read length of Illumina SBS technology, the Oxford nanopore sequencing results in longer read length at a lower cost with no amplification artifacts and bias [149]. Furthermore, the entire workflow for Clear Dx SARS-CoV-2 Test starting from cDNA synthesis to result interpretation, which takes 8 to 9 h to complete, is fully automated on the Clear Dx system that houses a robotic liquid handling platform, thermal cyclers, magnetic block and Oxford Nanopore GridION sequencer (Oxford Nanopore Technologies, Oxford, UK). The number of hands-on steps is reduced down to the set-up of the Clear Dx system and manual RNA extraction. Once the 96-well PCR plate containing extracted RNA is loaded into the system, the automated process will begin with the synthesis of cDNA followed by a multiplex PCR to capture viral target amplicon with a panel of 21 barcoded primers. Excess primers and short amplification products are then removed before the target amplicons are barcoded again in another round of PCR. The dual-barcoded amplicons from all the samples are pooled, clean-up and ligated with sequencing adapters to create sequencing libraries that are then loaded into the MinION flow cell and sequenced on the GridION sequencer.

Within the flow cell, protein nanopores are embedded in an electrically resistance polymer membrane with an ionic current passing through the nanopores. When a strand of DNA passes through a nanopore, the ionic current will be disrupted. As each of the DNA base causes a characteristic disruption in the current, the DNA sequence can be determined by measuring the change in current. Once sequencing has completed, the Clear Dx BIP is initiated and the bioinformatics pipeline includes demultiplexing, correction of errors, alignments of sequencing reads and detection calls. An invalid, positive or negative call is made based on a SARS-CoV-2 detection algorithm that takes into account the relative ratios of sequencing signals arising from SARS-CoV-2 primers, internal PCR control and housekeeping gene. The Clear Dx system can process up to 192 samples including positive and negative run controls. The Clear Dx SARS-CoV-2 Test demonstrated 100% PPA (95% CI: 92.9–100%) and NPA (95% CI: 89.3–100%) but has a higher LoD (2000 copies/mL) as compared to Illumina COVIDSeq Test (500–1000 copies/mL) [143,148].

## 14. Comparison of SARS-CoV-2 Reference Panel Results

Various factors may influence the selection of FDA-EUA NATs including the acceptable specimen type(s), complexity, cost, instrument and material requirements, turnaround time and the performance characteristics. In particular, the LoD can be a key determinant in test selection because a test will produce false negative results if the viral load of the specimens is lower than the LoD of the test. Hence, negative NAT result should not be used as the sole basis in ruling out the possibility of SARS-CoV-2 infection and must be combined with clinical observations, patient history and epidemiological information when determining a patient’s infection status. To reduce the risk of misdiagnosis, especially among specimens with low viral loads, a test with lower LoD is more sensitive and may be preferable over another with a higher LoD. However, the LoDs reported in the IFU/EUA summaries of FDA-EUA tests are not readily comparable because non-standardized materials and concentration units were used for LoD reporting. In order to facilitate the direct comparison of analytical sensitivity between different NAATs and to evaluate cross-reactivity with MERS-CoV virus, the FDA SARS-CoV-2 Reference Panel was developed and sent to the developers of 161 authorized assays as of 23 September 2020 [150]. The resulting sensitivity mean estimates of the EUA authorized SARS-CoV-2 tests are reported in NAAT detectable units/mL (NDU/mL) and reflects the extraction/instrument combination with the least sensitive LoD. The FDA categorizes these results in accordance with the clinical matrix that was used to conduct the testing (swab in transport media, direct swabs or saliva) [150].

**Figure 2:**
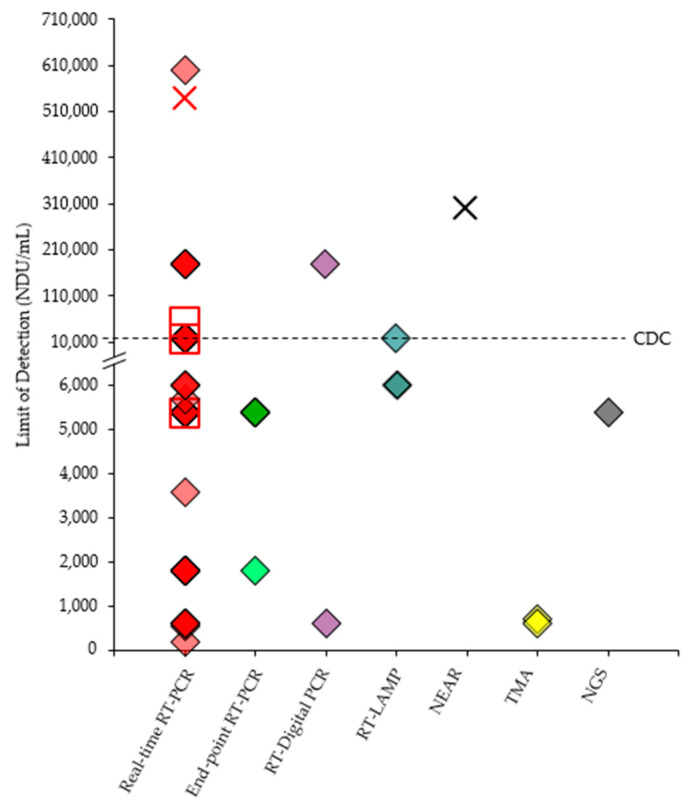
Sensitivity of FDA-EUA NATs based on SARS-CoV-2 Reference Panel Results. The clinical matrix used during testing were either swab in transport media (closed diamond), direct swab (cross) or saliva (open square). Limit of Detection (LoD) of the CDC 2019-Novel Coronavirus (2019-nCoV) Real-Time RT-PCR Diagnostic Panel (18,000 NDU/mL) is indicated by a horizontal dash line. NDU/mL, NAAT detectable units/mL.

Based on the available result of the FDA SARS-CoV-2 Reference Panel testing that was last updated on 6 November 2020, swabs in transport media that were tested with the RT-PCR platform resulted in the largest range of sensitivity (*n* = 56; 180–60,000 NDU/mL), as shown in Figure 2. PerkinElmer New Coronavirus Nucleic Acid Detection Kit is the most sensitive test and has a LoD of 180 NDU/mL whereas the least sensitive test is BMC-CReM COVID-19 Test with a LoD of 600,000 NDU/mL. Overall, isothermal amplification-based tests have similar or higher analytical sensitivity as compared to CDC 2019-nCoV Real-Time RT-PCR Diagnostic Panel (18,000 NDU/mL). These include the RT-LAMP-based tests such as the Color Genomics SARS-CoV-2 RT-LAMP Diagnostic Assay (18,000 NDU/mL), AQ-TOP COVID-19 Rapid Detection Kit (6000 NDU/mL), and Sherlock CRISPR SARS-CoV-2 Kit (6000 NDU/mL) as well as TMA-based, LetsGetChecked Coronavirus (COVID-19) Test (720 NDU/mL) and Aptima SARS-CoV-2 assay (600 NDU/mL). With a LoD of 5400 NDU/mL, the NGS-based Illumina COVIDSeq Test is also more sensitivity then the CDC 2019-nCoV Real-Time RT-PCR Diagnostic Panel. As for direct swab (dry swab) testing, the NEAR-based ID NOW COVID-19 (300,000 NDU/mL) demonstrated higher analytical sensitivity than the real-time RT-PCR-based Lyra Direct SARS-CoV-2 Assay (540,000 NDU/mL). LoDs that were established using saliva clinical matrix revealed that CRL Rapid Response (5400 NDU/mL) was the most sensitive followed by SalivaDirect (18,000 NDU/mL) and Advanta Dx SARS-CoV-2 RT-PCR Assay (54,000 NDU/mL). Of note, cross-reactivity with MERS-CoV was only observed in Phosphorus COVID-19 RT-qPCR Test. The availability of these information in the publicly accessible FDA website would greatly aid potential healthcare providers and patients to make informed choices relating to SARS-CoV-2 testing.

## 15. Future Perspectives and Conclusion

The resurgence of COVID-19 cases in many countries is a stark reminder that the pandemic has yet to be successfully brought under controlled and the number of cases around the world is still on the rise. Whilst the search for safe and effective therapeutics and vaccines is still ongoing, one of the key containment strategies is to correctly triage and identify COVID-19 patients at first point of contact. Therefore, POC technologies that can be deployed in the field for mass testing are in great demand as infected individuals among the general public can be rapidly identified, quarantined and treated in order to suppress human-to-human transmission. At present, manually-operated NATs are the predominant test format in the market as opposed to automated laboratory- and POC-based NATs [151]. With more than half of the authorized NATs being real-time RT-PCR tests, their availability is undeniably important for the initial expansion of testing capability as real-time systems are more readily found in most diagnostic laboratories as compared to other highly specialized and costly instruments such as electrochemical, magnetic resonance, MALDI-TOF and sequencing systems. Regardless of the amplification and detection technologies used, the hands-on and sample-to-result time of manual or semi-automated FDA-EUA NATs may also increase disproportionately with the increasing number of samples, especially when the tests require multiple manual liquid handling steps. FDA-EUA NATs that are designed to be run on fully automated systems can significantly reduce the reliance on highly trained personnel, shorten the turnaround time and increase the efficiency of the diagnostic testing workflow but the cost involved in purchasing, running and maintaining such instruments can be prohibitive to smaller laboratories. Nevertheless, diversification of the technological platform used for SARS-CoV-2 testing is deem vital, as it would allow laboratories to capitalize on their existing equipment and to select the test(s) that is/are best suited to the laboratories’ needs, expectations, capabilities and resources.

Presently, most of the FDA-EUA NATs are limited to CLIA-certified, high-complexity laboratories and require trained personnel to operate due to the technical intricacy associated with molecular tests. Although conventional RT-PCR tests take an average of 2 h to generate results, the turnaround time can be significantly extended due to the time taken for collection and transportation of patient specimens to a centralized lab, batched testing, generation of laboratory report and delivery of the report back to the doctor’s office for patient follow-up and notification [16]. The delay between infection and illness onset before a symptomatic patient seeks for professional help in addition to the time elapsed between sample collection to laboratory result generation means that it may take up to two weeks to receive a laboratory diagnosis [152]. As such, the development of POC-based NATs represents one of the major focus of future work as only seven NATs have received authorization for use under patient-care settings as of 31 October 2020 following the fulfilment of CLIA statutory criteria for waiver [60]. To determine the complexity of a test, the FDA uses a scorecard containing seven criteria: (1) knowledge; (2) training and experience; (3) reagents and materials preparation; (4) characteristics of operational steps; (5) calibration, quality control, and proficiency testing materials; (6) test system troubleshooting and equipment maintenance; and (7) interpretation and judgement. For each criterion, a score of one and three indicate the lowest and highest level of complexity, respectively. If a test is categorized as moderate complexity (total score ≤ 12), a CLIA waiver by application may be submitted to the FDA to request categorization of the test as waived.

Development of low-complexity POC NATs that are accurate, rapid and scalable for mass testing is critical during this pandemic as these tests would allow not only symptomatic but also asymptomatic cases to be diagnosed in a timely manner. POC diagnoses will also aid in tailoring public health interventions aimed at minimizing exposure to others, such as the duration of isolation and quarantine to be undertaken by COVID-19 cases, particularly among those with mild symptoms or who are asymptomatic. Ideally, the POC device should require very minimal manual manipulations by incorporating automated sample processing, amplification, detection and result interpretation steps. Microfluidic-based devices that are amenable to miniaturization is a highly attractive technological platform to achieve rapid POC diagnosis of COVID-19 due to its small sample volume requirement and high portability. We foresee that more POC NATs will become available in the near future and some of these are summarized in Table 2. At the time of writing, several POC NATs are known to be in development, including those by QuantuMDx Group Ltd. (Newcastle, UK), Scope Fluidics (Warsaw, Poland), binx health, Inc. (Boston, MA, USA), genedrive plc (Manchester, UK), Spartan Bioscience Inc. (Ottawa, Canada), Ubiquitome Ltd. (Auckland, New Zealand), MicroGEM International PLC (Southampton, UK), MatMaCorp (Materials and Machines Corporation) (Lincoln, OR, USA), Bosch (Waiblingen, Germany), and Talis Biomedical Corp (Menlo Park, CA, USA) [153,154,155,156,157,158,159].

Biosensors can also be a highly viable diagnostic platform to achieve rapid, ultra-sensitive and specific detection of SARS-CoV-2 given the recent advancements that have been made in the field of biosensor technology. A nucleic acid-based biosensor generally consists of two main components: a biological recognition element and a transducer. Once the target nucleic acids have been captured by the biological recognition element, the recognition event will be converted into a measurable signal by a transducer that can either be electrochemical, optical or piezoelectrical in nature. Biosensors have been shown to be capable of attaining detection limits in the fentomolar and attomolar range by tapping into the various types of signal amplification strategies. For a more comprehensive discussion on the potential application of different biosensing approaches for the detection of SARS-CoV-2, the review by Samson et al. [167] is recommended. Biosensors at the proof-of-concept stage provide a glimpse of their potentials to revolutionize diagnostic testing, but the journey to commercialization remains long, costly and arduous. As a molecular diagnostics company with its own proprietary sensor technology, GenMark Diagnostics was able to offer a biosensor-based NAT for SARS-CoV-2 detection in a relatively short period of time as the established eSensor technology can be easily adapted by modifying the primers and probes used.

In conclusion, development of NATs for mass testing and for use at POC settings should take precedence given the limited number of such tests at this point of time. While the use of authorized NATs provide assurance that the tests are accurate and reliable, the performance of NATs can be adversely affected by multiple factors as discussed in this review. The shortage of essential consumables in the molecular diagnostic workflow that has become a severe bottleneck to widespread testing is one of the pressing issues that need to be resolved with innovative NAT solutions that can help to ease the strain on the supply chain. With the arrival of the second wave of COVID-19 that has already struck several parts of the world, research and development of nucleic acid-based diagnostic tests that can lead to early identification and isolation of cases, along with prompt contact tracing, will continue to be paramount in the fight against the spread of COVID-19.

## Figures and Tables

**Table 1:** Comparison of various FDA-EUA NATs for the detection of SARS-CoV-2.

Developer	Name of the Test	Authorized Setting	Targeted Gene(s) of SARS-CoV-2	Limit of Detection (LoD)
Real-Time RT-PCR *				
Beijing Wantai Biological Pharmacy Enterprise Co., Ltd.	Wantai SARS-CoV-2 RT-PCR Kit	H	*Orf1ab*, *N*	50 copies/mL
Fluidigm Corporation	Advanta Dx SARS-CoV-2 RT-PCR Assay	H	*N1*, *N2*	6.25 GE/µL
Yale School of Public Health, Department of Epidemiology of Microbial Diseases	SalivaDirect	H	*N1*	6 copies/µL
Biomeme, Inc.	Biomeme SARS-CoV-2 Real-Time RT-PCR Test	H	*Orf1ab*, *S*	1.8 GE/µL
Jiangsu CoWin Biotech Co., Ltd.	Novel Coronavirus (SARS-CoV-2) Fast Nucleic Acid Detection Kit (PCR-Fluorescence Probing)	H	*Orf1ab*, *N*	300 copies/mL
Access Bio, Inc.	CareStart COVID-19 MDx RT-PCR	H	*N*, *RdRp*	10 copies/reaction
Centers for Disease Control and Prevention (CDC)	Influenza SARS-CoV-2 (Flu SC2) Multiplex Assay	H	*N*	1.01 × 10^−2^ TCID_50_/mL (TaqPath 1-step Multiplex); 5.06 × 10^−2^ TCID_50_/mL (Ultraplex 1-step ToughMix)
RTA Laboratories Biological Products Pharmaceutical and Machinery Industry	Diagnovital SARS-CoV-2 Real-Time PCR Kit	H	*E*, *RdRp*	38 copies/mL
dba SpectronRX	Hymon SARS-CoV-2 Test Kit	H	*E*, *N*	5 copies/µL
Quidel Corporation	Lyra Direct SARS-CoV-2 Assay	H	*pp1ab*	1.28 × 10^4^ GE/mL
GeneMatrix, Inc.	NeoPlex COVID-19 Detection Kit	H	*N*, *RdRp*	50 copies/reaction
BioMérieux SA	SARS-COV-2 R-GENE	H	PCR1: *N, RdRp* PCR2: *E*	380 GC/mL
Sansure BioTech Inc.	Novel Coronavirus (2019-nCoV) Nucleic Acid Diagnostic Kit (PCR-Fluorescence Probing)	H	*Orf1ab*, *N*	200 copies/mL
Thermo Fisher Scientific, Inc.	TaqPath COVID-19 Combo Kit	H	*Orf1ab*, *S*, *N*	10 GCE/reaction
Wadsworth Center, NYSDOH	New York SARS-CoV-2 Real-time Reverse Transcriptase (RT)-PCR Diagnostic Panel	H	*N1*, *N2*	25 GC/reaction
Centers for Disease Control and Prevention’s (CDC)	CDC 2019-Novel Coronavirus (2019-nCoV) Real-Time RT-PCR Diagnostic Panel	H	*N1*, *N2*	10^0^ RNA copies/µL (Qiagen DSP); 10^0.5^ RNA copies/µL (Qiagen EZ1)
**Real-time RT-PCR for Use on Automated Sample-to-result System**				
BioFire Diagnostics, LLC	BioFire Respiratory Panel 2.1-EZ (RP2.1-EZ)	H, M, W	*S*, *M*	5 × 10^2^ copies/mL
Cepheid	Xpert Xpress SARS-CoV-2/Flu/RSV	H, M, W	*E*, *N2*	131 copies/mL
Roche Molecular Systems, Inc.	cobas SARS-CoV-2 & Influenza A/B Nucleic Acid Test for use on the cobas Liat System	H, M, W	*Orf1a/b*, *E*	12 copies/mL
Roche Molecular Systems, Inc.	cobas SARS-CoV-2 & Influenza A/B	H, M	*Orf1a/b*, *E*	0.12 TCID_50_/mL
BayCare Laboratories, LLC	BayCare SARS-CoV-2 RT PCR Assay	H	*ORF1*, *E*	0.007 TCID_50_/mL
Kaiser Permanente Mid-Atlantic States	KPMAS COVID-19 Test	H	*Orf1ab*, *E*	0.007 TCID_50_/mL (Orf1ab); 0.004 TCID_50_/mL (E)
University of California San Diego Health	UCSD RC SARS-CoV-2 Assay	H	*Orf1ab*, *E*	0.007 TCID_50_/mL (Orf1ab); 0.004 TCID_50_/mL (E)
Quest Diagnostics Infectious Disease, Inc.	Quest Diagnostics PF SARS-CoV-2 Assay	H	2 regions of *Orf1ab*	1 × 10^−2^ TCID_50_/mL
Quest Diagnostics Infectious Disease, Inc.	Quest Diagnostics RC SARS-CoV-2 Assay	H	*Orf1ab*, *E*	0.007 TCID_50_/mL (Orf1ab); 0.004 TCID_50_/mL (E)
Omnipathology Solutions Medical Corporation	Omni COVID-19 Assay by RT-PCR	H	*N1*, *N2*	1.23 copies/µL
Abbott Molecular Inc.	Alinity m SARS-CoV-2 assay	H, M	*N*, *RdRp*	100 virus copies/mL
BioFire Diagnostics, LLC	BioFire Respiratory Panel 2.1 (RP2.1)	H, M	*S*, *M*	5.0 × 10^2^ copies/mL
Becton, Dickinson & Company (BD)	BD SARS-CoV-2Reagents for BD MAX System	H, M	*N1*, *N2*	40 GE/mL
Luminex Corporation	ARIES SARS-CoV-2 Assay	H, M	*Orf1ab*, *N*	3.33 × 10^2^ GCE/mL
Becton, Dickinson & Company (BD)	BioGX SARS-CoV-2 Reagents for BD MAX™ System	H, M	*N1*, *N2*	40 GE/mL
QIAGEN GmbH	QIAstat-Dx Respiratory SARS-CoV-2 Panel	H, M	*Orf1b*, *E*	500 copies/mL
NeuMoDx Molecular, Inc.	NeuMoDx SARS-CoV-2 Assay	H, M	*N*, *Nsp2*	150 copies/mL
BioFire Defense, LLC	BioFire COVID-19 Test	H, M	2 regions of *Orf1ab*, *Orf8*	3.3 × 10^2^ GC/mL
Cepheid	Xpert Xpress SARS-CoV-2	H, M, W	*E*, *N2*	0.02 PFU/mL
DiaSorin Molecular LLC	Simplex COVID-19 Direct	H, M	*Orf1ab*, *S*	500 copies/mL
Abbott Diagnostics Scarborough, Inc.	Abbott RealTime SARS-CoV-2	H	*N*, *RdRp*	100 virus copies/mL
Hologic, Inc.	Panther Fusion SARS-CoV-2 Assay	H	2 regions of *Orf1ab*	1 × 10^−2^ TCID_50_/mL
Roche Molecular Systems, Inc.	cobas SARS-CoV-2	H, M	*Orf1ab*, *E*	0.007 TCID_50_/mL (Orf1ab); 0.004 TCID_50_/mL (E)
**End-point RT-PCR with Lateral Flow Detection**				
Mesa Biotech Inc.	Accula SARS-CoV-2 Test	H, M, W	*N*	200 copies/reaction
**End-point RT-PCR with Enzyme-based Colorimetric Detection**				
Visby Medical, Inc.	Visby Medical COVID-19	H, M	*N*	1112 GC/mL
Rheonix, Inc.	Rheonix COVID-19 MDx Assay	H	*N1*	625 GE/mL
**End-point RT-PCR with Fluorescence Detection**				
Alimetrix, Inc.	Alimetrix SARS-CoV-2 RT-PCR Assay	H	*Orf1ab*, *N1*, *N2*	250 copies/mL (Zymo Research Quick-DNA/RNA Viral MagBead Extraction); 1000 copies/mL (Qiagen QIAamp 96 Virus QIAcube HT Kit)
DxTerity Diagnostics, Inc.	DxTerity SARS-CoV-2 RT PCR CE Test	H	*Orf1ab*, *E*, *N*	50 copies/mL
QDx Pathology Services	QDX SARS-CoV-2 Assay	H	*N1*, *N2*	1000 copies/mL (Applied Biosystems 7500 Fast and the Applied Biosystems Quant Studio 7 systems), 250 copies/mL (Applied Biosystems Quant Studio 12K system)
PlexBio Co., Ltd.	IntelliPlex SARS-CoV-2 Detection Kit	H	*E*, *N*, *RdRp*	140 copies/mL
Applied BioCode, Inc.	BioCode SARS-CoV-2 Assay	H	2 regions in *N* gene	1.72 × 10^−2^ TCID_50_/mL
ChromaCode Inc.	HDPCR SARS-CoV-2 Assay	H	*N1*, *N2*	1000 copies/mL (Applied Biosystems 7500 Fast and the Applied Biosystems Quant Studio 7 systems), 250 copies/mL (Applied Biosystems Quant Studio 12K system)
**End-point RT-PCR with Electrochemical Detection**				
GenMark Diagnostics, Inc.	ePlex Respiratory Pathogen Panel 2	H, M	NR	250 GC/mL
GenMark Diagnostics, Inc.	ePlexSARS-CoV-2 Test	H, M	NR	750 GC/mL
**End-point RT-PCR with Magnetic Resonance Detection**				
T2 Biosystems, Inc.	T2SARS-CoV-2 Panel	H, M	NR	2000 GE/mL
**End-point RT-PCR with MALDI-TOF Detection**				
Agena Bioscience, Inc.	MassARRAY SARS-CoV-2 Panel	H	*Orf1ab*, *N1*, *N2*, *N3*, *ORF1*	2.5 copies/μL
National Jewish Health	SARS-CoV-2 MassArray Test	H	*Orf1ab*, *N1*, *N2*, *N3*, *ORF1*	0.69 copies/µL
Ethos Laboratories	Ethos Laboratories SARS-CoV-2 MALDI-TOF Assay	H	*Orf1ab*, *N1*, *N2*, *N3*, *ORF1*	1 TCID_50_/mL
**RT-Digital PCR**				
PreciGenome LLC	FastPlex Triplex SARS-CoV-2 detection kit (RT-Digital PCR)	H	*Orf1ab*, *N*	571.4 copies/mL
Gnomegen LLC	Gnomegen COVID-19 RT-Digital PCR Detection Kit	H	*N1*, *N2*	8 GC/reaction
Bio-Rad Laboratories, Inc	Bio-Rad SARS-CoV-2 ddPCR Test	H	*N1*, *N2*	150 copies/mL
**qSTAR**				
LumiraDx UK Ltd.	LumiraDx SARS-CoV-2 RNA STAR Complete	H	*Orf1a*	7500 copies/mL
LumiraDx UK Ltd.	LumiraDx SARS-CoV-2 RNA STAR	H	*Orf1a*	500 copies/mL
**Isothermal Nucleic Acid Amplification**				
Cue Health Inc.	Cue COVID-19 Test	H, M, W	*N*	1.3 GC/µL
**RT-LAMP with Fluorescence Detection**				
Seasun Biomaterials, Inc.	AQ-TOP COVID-19 Rapid Detection Kit PLUS	H	*Orf1ab*, *N*	1 copy/µL
Pro-Lab Diagnostics	Pro-AmpRT SARS-CoV-2 Test	H	*Orf1ab*	125 GE/swab
Seasun Biomaterials, Inc.	AQ-TOP COVID-19 Rapid Detection Kit	H	*Orf1ab*	7 copies/µL
**RT-LAMP with Colorimetric Detection**				
Detectachem Inc.	MobileDetect Bio BCC19 (MD-Bio BCC19) Test Kit	H, M	*E*, *N*	75 copies/µL
Color Genomics, Inc.	Color Genomics SARS-CoV-2 RT-LAMP Diagnostic Assay	H	*Orf1ab*, *E*, *N*	0.75 copies/µL
**RT-LAMP with CRISPR-based Detection**				
Mammoth Biosciences, Inc.	SARS-CoV-2 DETECTR Reagent Kit	H	*N*	20 copies/µL
UCSF Health Clinical Laboratories, UCSF Clinical Labs at China Basin	SARS-CoV-2 RNA DETECTR Assay	H	*N*	20 copies/µL
Sherlock BioSciences, Inc.	Sherlock CRISPR SARS-CoV-2 Kit	H	*Orf1ab*, *N*	6.75 copies/µL (Orf1ab); 1.35 copies/µL (N)
**NEAR**				
Abbott Diagnostics Scarborough, Inc.	ID NOW COVID-19	H, M, W	*RdRp*	125 GE/mL
**OMEGA Amplification**				
Atila BioSystems, Inc.	iAMP COVID-19 Detection Kit	H	*Orf1ab*, *N*	10 copies/μL
**TMA**				
Poplar Healthcare	Poplar SARS-CoV-2 TMA Pooling assay	H	2 regions of *Orf1ab*	83 copies/mL
Quest Diagnostics Infectious Disease, Inc.	Quest Diagnostics HA SARS-CoV-2 Assay	H	2 regions of *Orf1ab*	83 copies/mL
PrivaPath Diagnostics, Inc.	LetsGetChecked Coronavirus (COVID-19) Test	H	2 regions of *Orf1ab*	83 copies/mL
Hologic, Inc.	Aptima SARS-CoV-2 assay	H	2 regions of *Orf1ab*	83 copies/mL
**Sanger Sequencing**				
BillionToOne, Inc.	qSanger-COVID-19 Assay	H	*N*	3200 copies/mL
**NGS**				
University of California, Los Angeles (UCLA)	UCLA SwabSeq COVID-19 Diagnostic Platform	H	*S2*	250 GCE/mL
Clear Labs, Inc.	Clear Dx SARS-CoV-2 Test	H	21 target genes	2000 copies/mL
Guardant Health, Inc.	Guardant-19	H	*N1*	125 copies/mL
Helix OpCo LLC (dba Helix)	Helix COVID-19 NGS Test	H	*S*	125 GCE/mL
Illumina, Inc.	Illumina COVIDSeq Test	H	98 target genes	1000 copies/mL

* A representative selection of real-time RT-PCR tests are shown here (Please refer to Table S1 for complete detail). H, CLIA-certified high complexity lab; M, CLIA-certified medium complexity lab; W, CLIA-waived tests; GE, genomic equivalents; GC, genomic copies; GCE, genomic copy equivalents; PFU, plaque forming units; NR, not reported.

**Table 2:** Emerging point-of-care (POC) NATs for the detection of SARS-CoV-2.

Developer	Name of Test	Technology	Specimen Indicated for Testing	Time to Result	Limit of Detection	Target Gene	Status	Reference
DnaNudge Ltd.	CovidNudge Test	RT-PCR	NP swab or sputum	90 min	250 viral copies/swab	*N1*, *N2*, *N3*, *RdRp1*, *RdRp2*, *E*	CE-IVD	[160]
Diagnostics for the Real World Ltd.	SAMBA II SARS-CoV-2 Test	Isothermal amplification, lateral flow	Throat and nose swabs	<90 min	250 copies/mL	*Orf1ab*, *N*	CE-IVD	[161]
OptiGene Ltd.	COVID-19_Direct Plus RT-LAMP KIT-500 kit	RT-LAMP	OP/NP swab dilutions and saliva samples	<20 min	10^3^ copies/mL	NR	CE-IVD	[162]
Caspr Biotech	Caspr Lyo-CRISPR SARS-CoV-2 Kit (FAM) Direct Sample	RT-LAMP, CRISPR	NP/OP and nasal swabs	<60 min	25 copies/μL	2 regions in *N*, *Orf1ab*	N/A	[163]
Molbio Diagnostics Pvt Ltd.	Truenat SARS CoV-2	Chip-based Real-Time RT-PCR	OP and NP swabs	35 min	407 genome copies/mL	*RdRp*	India CDSCO	[164]
Mobidiag	Novodiag COVID-19	RT-PCR	NP swab	1 h 20 min	NR	*Orf1ab*, *N*	CE-IVD	[165]
GeneReach Biotechnology Corp	POCKIT Central SARS-CoV-2 (*Orf1ab*) Premix Reagent	Insulated Isothermal PCR (iiPCR) technology	OP swab	85 min	NR	*Orf1ab*	CE-IVD	[166]

NP, nasopharyngeal; OP, oropharyngeal; NR, not reported.

## Supplementary Materials

The following are available online at https://www.mdpi.com/2075-4418/11/1/53/s1.
